# The C-terminal domain of the type III secretion chaperone HpaB contributes to dissociation of chaperone-effector complex in *Xanthomonas campestris* pv. *campestris*

**DOI:** 10.1371/journal.pone.0246033

**Published:** 2021-01-28

**Authors:** Yong-Liang Gan, Li-Yan Yang, Li-Chao Yang, Wan-Lian Li, Xue-Lian Liang, Wei Jiang, Guo-Feng Jiang, Xiao-Hong Hang, Mei Yang, Ji-Liang Tang, Bo-Le Jiang

**Affiliations:** 1 State Key Laboratory for Conservation and Utilization of Subtropical Agro-bioresources, College of Life Science and Technology, Guangxi University, Nanning, China; 2 College of Forest, Guangxi University, Nanning, China; 3 School of Chemistry and Chemical Engineering, Guangxi University, Nanning, China; Shanghai Jiao Tong University, CHINA

## Abstract

Many animal and plant pathogenic bacteria employ a type three secretion system (T3SS) to deliver type three effector proteins (T3Es) into host cells. Efficient secretion of many T3Es in the plant pathogen *Xanthomonas campestris* pv. *campestris* (*Xcc*) relies on the global chaperone HpaB. However, how the domain of HpaB itself affects effector translocation/secretion is poorly understood. Here, we used genetic and biochemical approaches to identify a novel domain at the C-terminal end of HpaB (amino acid residues 137–160) that contributes to virulence and hypersensitive response (HR). Both *in vitro* secretion assay and *in planta* translocation assay showed that the secretion and translocation of T3E proteins depend on the C-terminal region of HpaB. Deletion of the C-terminal region of HpaB did not affect binding to T3Es, self-association or interaction with T3SS components. However, the deletion of C-terminal region sharply reduced the mounts of free T3Es liberated from the complex of HpaB with the T3Es, a reaction catalyzed in an ATP-dependent manner by the T3SS-associated ATPase HrcN. Our findings demonstrate the C-terminal domain of HpaB contributes to disassembly of chaperone-effector complex and reveal a potential molecular mechanism underpinning the involvement of HpaB in secretion of T3Es in *Xcc*.

## Introduction

Plant pathogenic *Xanthomonas* utilizes a conserved type three secretion system (T3SS) to promote growth and disease in host plants [1]. The secretion/translocation of type three effector (T3E) proteins into host cells via the T3SS allows the pathogen to hijack and overcome the defense responses of the host [2,3]. The T3SS of *Xanthomonas* is encoded by a chromosomal *hrp* [hypersensitive response (HR) and pathogenicity] cluster regulated by two master regulators, HrpG and HrpX, whose expression is highly elevated *in planta* or in particular minimal media [4–7]. The T3SS of *Xanthomonas* is a molecular nanomachine and consists of peripheral cytoplasm components (HrcQNL and HrpB7), inner membrane (IM) components (HrcUVRST), periplasm or associated IM components (HrcJ, HrpB1, and HrpB2), outer membrane (OM) component (HrcC), extracellular Hrp pili (HrpE), and translocon (HrpF) [1,8–14].

The efficient assembly of the T3SS and secretion of effectors also depend upon chaperones which are divided into different classes according to their substrate specificities: class I bind to effectors, class II bind to translocon or pore-forming proteins, and class III bind to needle and filament proteins [1,15]. Class I chaperones are sub-divided into class IA, which bind to single or several homologous effectors and class IB, which bind to several effectors. It has been established that secretion/translocation of a number of T3Es of *Xanthomonas* and *Ralstonia* depends on the T3S chaperone HpaB [16–20] which belongs to class IB, and this class chaperones generally target the N terminal regions of T3Es termed chaperone-binding domains (CBDs) [1,21]. Although in the majority of cases, CBDs are located within the N-terminal regions of T3Es, some variations do occur. For example, the CBD of the T3E HpaA in *Xanthomonas* has been located in its C terminal region [22].

The interaction of T3Es with chaperones allows presentation of the complex to the secretion apparatus prior to translocation. Recent work has shown that T3Es of *X*. *campestris* pv. *vesicatoria* (*Xcv*), prior to interaction with HpaB, locate to the bacterial cytoplasmic membrane by specifically binding to cardiolipin [23]. Several studies in animal pathogenic bacteria have shown that the T3E wraps around the chaperone dimer in an extended conformation before targeting to the secretion platform, leading to the proposal that there is a three-dimensional signal for T3SS recognition [1,15,24–26]. As the T3S chaperone cannot be secreted or translocated, it must be released from chaperone-effector complex before effector secretion. This action is performed by a T3S-associated ATPase (InvC in *Salmonella enterica* and HrcN in *Xanthomonas*) in an ATP-dependent manner [11,27]. The T3S-associated ATPase also functions as unfoldase for T3Es, keeping an unfolded or partial-unfolded conformation for competent secretion [27,28].

Thus far, the detailed molecular mechanisms for dissociation of chaperone-effector complexes by a T3S-associated ATPase have remained unclear. In this study, we examined the role of T3S chaperone HpaB of *Xanthomonas campestris* in more detail. We show that the C terminal region of this protein (amino acid residues 137–160, hereafter referred as C-terminal domain) are required for both virulence and HR in *Xanthomonas*. An HpaB variant with a C-terminal truncation can bind to T3Es but is hardly released by HrcN in an ATP-dependent manner, resulting in a blockage of the action of the T3SS.

## Materials and methods

### Bacterial strains, plasmids, and growth conditions

The bacteria and plasmids used in this study are listed in S1 Table. *Escherichia coli* strains were grown in LB medium [29] at 37°C or 16°C (for protein expression). *Xanthomonas* strains were cultured in a complete NYG medium or in MMX (minimal medium for *Xanthomonas campestris*) medium at 28°C [30]. Plasmids were introduced into *E*. *coli* strains by electroporation and into *Xanthomonas* strains by triparental conjugation or electroporation. Antibiotics were supplemented to media in the following final concentrations: ampicillin (Amp), 100 μg/ml; kanamycin (Kan), 25 μg/ml; spectinomycin (Spc), 50 μg/ml; tetracycline (Tc), 15 μg/ml for *E*. *coli* strains and 5 μg/ml for *Xanthomonas* strains; rifampicin (Rif), 50 μg/ml.

### Construction of *hpaB* deletion mutants and complementation

To generate a full-length deletion of *hpaB* gene, two about 700-bp-long flanking sequences of *hpaB* were amplified with corresponding primer pair (S2 Table) and linked into a suicide plasmid pK18mobsacB resulting in a recombinant plasmid pK18-ΔhpaB (S1 Table). The pK-ΔhpaB was introduced into *Xcc* strain to generate ΔhpaB as described earlier [31]. For deletion of both residues 45–160 and 137–160 of HpaB, similar methods were performed as mentioned above.

For complementation of *hpaB* deletion mutants, a 1083-bp DNA fragment containing 500 bp upstream, ORF, and 100 bp downstream of *hpaB* gene was cloned into pLAFRJ to generate a recombinant for complementation analysis.

### Plant assay

For virulence assay, *Xcc* strains were cultured in NYG medium at 28°C with shaking at 200 rpm overnight and adjusted to an OD_600_ of 0.001. Host plant Chinese radishes (*Raphanus sativus* var. *radiculus* cv. Manshenhong) were grown in a glasshouse with natural light and temperatures of 25–30°C. After transferring to greenhouse with a 12-h day and 12-h night cycle of illumination by fluorescent at 25–28°C, seedlings with four fully expanded leaves were used for inoculations by the leaf-clipping method [32]. At least 60 leaves were inoculated for each strain, and the experiment was repeated three times independently. After inoculation, the plants were maintained in 80% humidity for the first 24 h, and then in the conditions described above. The lesion lengths of the inoculated leaves were measured at 10 days post-inoculation, and the data were analyzed by *t*-test.

The HR induced by *Xcc* strains was tested on non-host pepper plants (*Capsicum annuum* cv. ECW-10R), which were grown in greenhouse with a 12-h day and 12-h night cycle of illumination by fluorescent at 28°C, with 80% relative humidity. The bacterial cells of *Xcc* strains from overnight cultures were washed, resuspended and diluted to an OD_600_ of 0.01 or 0.1 in double-distilled H_2_O, and infiltrated into the pepper leaf tissues at the stage of four fully expanded leaves using a needleless syringe. HR symptoms were photographed at 8–24 h post-inoculation. At least three plants were inoculated in each experiment. All experiments were repeated at least twice.

### T3S secretion assay

For *in vitro* secretion, ORFs of *XC2081* (*avrBs1*), *XC3176*, and *XC3002* (*hpa1*) were cloned into a host-broad plasmid pJXG containing a 3×FLAG-encoding sequence [18], respectively. Bacteria of *Xcc* were cultured in rich NYG medium overnight and were transferred into *hrp*-inducing medium MMX at same cell density for 8 h. The total cell extracts and cultural supernatants were isolated as described previously [31]. Equal amounts of total cell extract and cultural supernatants were separated by SDS-PAGE and analyzed by immunoblotting, using anti-Flag-tag rabbit monoclonal antibody (1/1000, Sigma). Horseradish peroxidase-conjugated goat anti-rabbit immunoglobulin G (IgG) (1/1000, Pierce) was used as secondary antibody. To ensure that no bacterial lysis had occurred, the protein blots were routinely probed with a rabbit monoclonal antibody specific for β subunit of *E*. *coli* RNA polymerase (RNP*β*, 1/1000, Abcam, ab12087). Reactions were visualized by enhanced chemiluminescence. All experiments were repeated at least twice.

### T3S translocation assay

To assess translocation efficiency of T3E, an AvrBs1-based reporter system and a calmodulin-dependent adenylate cyclase (Cya) reporter system were used [18,33,34]. Taking construction of pJAG1553 and pJAA1553 as an example, An 894 bp DNA fragment containing 588 bp upstream and first 102 codons of *XC1553* (*avrAC*) was amplified and linked to the reporter plasmid pJAG [18,35] harboring a DNA fragment that encodes AvrBs1_59-445_ responding to R gene *Bs1* of pepper line ECW-10R, and pJAA [36] harboring a *cya* gene DNA (encoding Cya_2-400_) from *Bordetella pertussis*, respectively. The resulting recombinant plasmid pJAG1553 and pJAA1553 were introduced into *Xcc* strains, respectively. HR was tested as mentioned above. The bacterial suspensions were diluted to an OD_600_ of 0.1 and infiltrated into cabbage (*Brassica oleracea* cv. Jingfeng NO.1) leaf tissues. To assay adenylate cyclase activity in plant tissue infected by bacteria, the cAMP content of triplicate samples was determined as described method [36]. All experiments were repeated at least twice.

### Overexpression and purification of proteins

For construction of GST-HpaB and its variants, the target fragments of *hpaB* were amplified using corresponding primer pairs (S2 Table) and cloned into the *Eco*RI/*Xho*I or *Bam*HI/*Eco*RI sites of pGEX-4T-1 in frame with *gst* gene. The amplified fragments of *hpaB* and its variants, *avrBs1*, *avrAC*, and *XC3176* were linked to the *Eco*RI/*Xho*I sites of pET30a and *hpaB* was also liked to the *Eco*RI/*Xho*I sites of pET32a. The resulting recombinant plasmids were introduced into *E*. *coli* BL21(DE3) or *E*. *coli* C43(DE3). Bacteria were grown in LB at 37°C. At OD_600_ of 0.5–0.7, protein productions were induced by addition of 0.1–0.5 mM isopropyl-*β*-D-1-thiogalactopyranoside (IPTG) at 20°C for 8–24 h. Cells were pelleted for 10 min at 4,000 g. Cell pellets were resuspended in TBS (20 mM Tris, 150 mM NaCl, pH 8.0) plus 1 mM phenylmethylsulfonyl fluoride (PMSF), and sonicated on ice. Broken cells were centrifuged at 20,000 g at 4°C for 20 min, and the supernatants were incubated with Glutathione Sepharose (GE, Uppsala, Sweden) or Ni Sepharose (GE, Uppsala, Sweden) for 1 h at 4°C. For purifying GST-fusion protein, the resin was washed with 10 column volume of TBS buffer and eluted with 10 mM reduced glutathione in TBS. For purifying His-fusion protein, the resin was washed with 50 column volume of high-salt TBS buffer (20 mM Tris, 500 mM NaCl, 10–20 mM imidazole, pH 8.0) and eluted by 250–500 mM imidazole in high-salt TBS buffer. To remove elution buffer, purified protein solutions were ultra-filtered and washed with TBS buffer.

### GST-pull down

For protein-protein interactions, 50 ml cell cultures of GST-fused HpaB proteins and GST alone were harvested by centrifugation, resuspended with binding buffer (20 mM Tris, 150 mM NaCl, 0.05% Triton-100, pH 8.0), and sonicated on ice. Cell debris was removed and equal amounts of soluble cell lysate were immobilized on 10 μl of MagneGST^TM^ particles (Promega, Madison, USA). The *E*. *coli* cell lysates expressing prey protein (6×His-tagged protein) were incubated with those immobilized proteins for 30 min at room temperature or overnight at 4°C, followed by washing unbound protein with binding buffer. After addition of 1×SDS-loading buffer, the binding proteins to magnetic beads were analyzed by SDS-PAGE and immunoblotting with anti-His mouse monoclonal antibody (1/5000, Abbkine) or anti-GST rabbit polyclonal antibody (1/4000, Proteintech). Horseradish peroxidase-conjugated goat anti-rabbit or -mouse immunoglobulin G (IgG) (1/1000, Pierce) were used as secondary antibodies. The experiments were performed more than two repeats.

### ATPase assay

ATPase activity of HrcN was determined as previously reported [11] with a few modifications. Briefly, 160 μl of reaction mixtures consisting of one microgram of purified 6×His-HrcN, 0–160 mM ATP (final concentration) in modified TBS buffer (20 mM Tris, 150 mM NaCl, 1mM MgCl_2_, pH 7.0) were incubated for 30 min at 37°C, colored with 100 μl of malachite green reagent at room temperature for 10 min, and stopped with 34% citric acid. The absorbance at 650 nm of triplicate samples was measured. For the calculation of the amounts of released phosphate, the absorbance of a phosphate standard curve was performed according to the manufacturer’s instructions.

### Chaperone release assay

Chaperone release assay was determined as previously reported [11]. Briefly, the GST-fused chaperone HpaB derivatives or GST alone were immobilized on MagneGST^TM^ particles, incubated with the T3Es (XC3176 or AvrAC) tagged by 6×His tag, and formed stable chaperone-effector complex. After removing of unbound proteins, the chaperone-effector complex immobilized on MagneGST^TM^ particles were aliquoted, and then 1 μg of 6×His-HrcN was added into the aliquots of reactive mixture in presence of 150 μM ATP (final concentration) and incubated at room temperature for 1 h. The released effector in solution was separated by magnet and analyzed by SDS-PAGE and immunoblotting.

## Results

### HpaB of *Xanthomonas campestris* pv. *campestris* is a global type III chaperone

HpaB from *Xanthomonas campestris pv*. *campestris* (*Xcc*) is a small protein of 160-amino acid which shares common physical characteristics of type III secretion chaperones, including an acidic pI (4.26), a predicted amphipathic helix near its C-terminal end (amino acid residues 118–139) and rich leucine content (13.6%) [37,38]. HpaB contains a predicted CesT domain (amino acid residues 8–136) (S1 Fig), which acts as a chaperone for the *E*. *coli* (EPEC or EHEC) translocated intimin receptor (Tir) protein [39,40].

**Fig 1:**
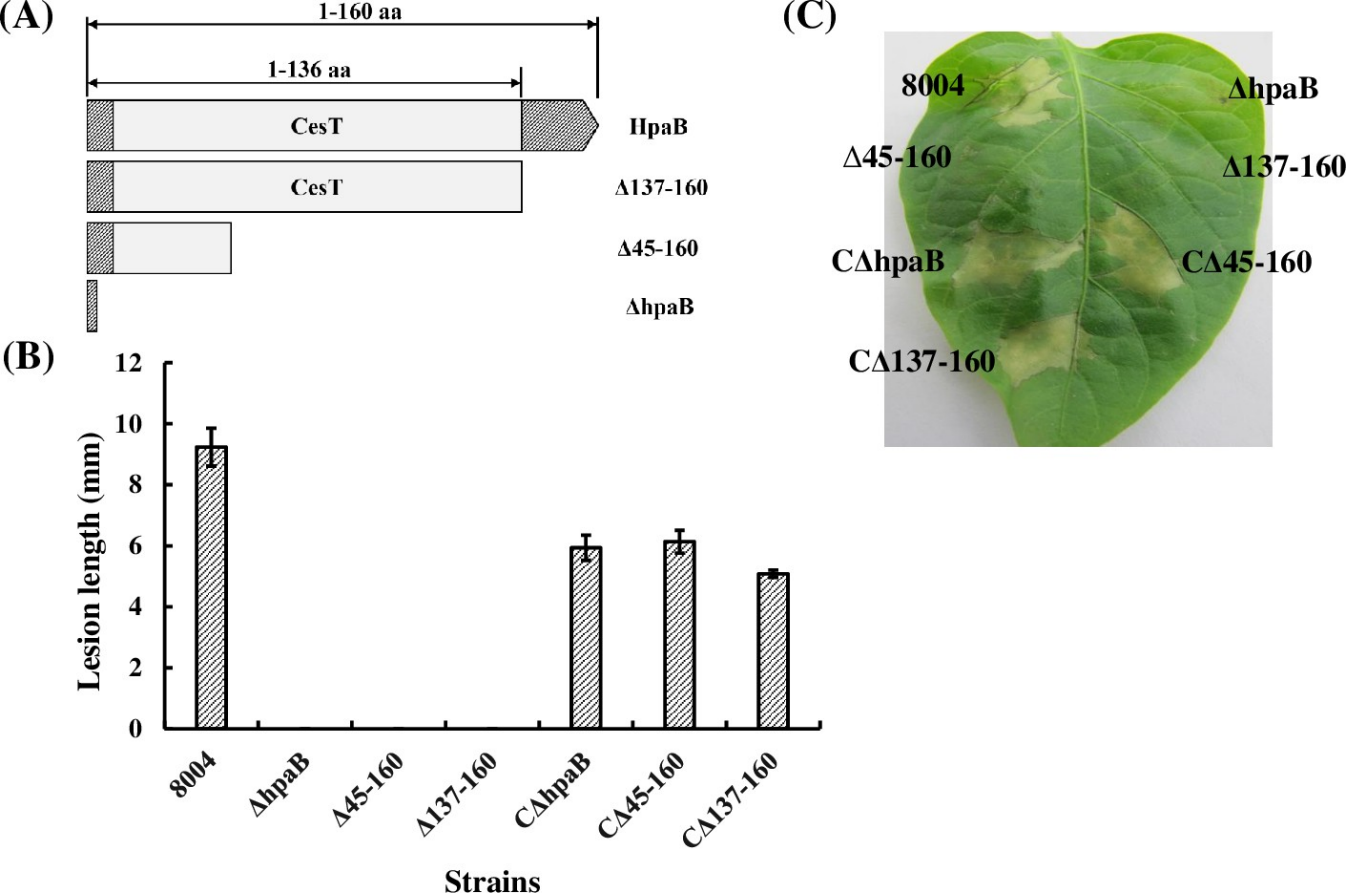
HpaB and its C-terminal domain are involved in virulence and HR induction in *Xcc*. (**A**) Schematic presentation of generation of *hpaB* deletion mutants. aa = amino acid residues. (**B**) The average lesion lengths of Chinese radish (*Raphanus sativus* var. *radiculus* cv. Manshenhong) leaves caused by *Xcc* strains (wild type strain 8004, *hpaB*-deletion mutants, and *hpaB*-deletion mutants carrying *hpaB*-recombinant plasmid) with a cell density of OD_600_ = 0.001 were measured 10 days post-inoculation. Values are the mean ± standard deviation from three repeats, each with at least 60 leaves. (**C**) Analysis of HR induced by *Xcc* strains. Bacteria of *Xcc* with a cell density of OD_600_ = 0.1 were inoculated into the leaves of pepper plant (*Capsicum annuum* cv. ECW-10R). Leaves were photographed at 24 h post-inoculation.

HpaB from *Xcv* is involved in bacterial virulence and HR induction [16]. Although HpaB from *Xcc* contributes to the elicitation of HR reaction in nonhost plants [18], its role in pathogenicity is unknown. To investigate this, a deletion strain of full-length *hpaB* was generated and named ΔhpaB (S1 Table). In addition, a 1083-bp DNA fragment comprising the *hpaB* gene and its promoter was cloned into pLAFRJ to generate a recombinant for complementation analysis. As shown in Fig 1, the ΔhpaB strain had a complete loss of virulence on the host plant Chinese radish (*Raphanus sativus* var. *radiculus* cv. Manshenhong) and was unable to cause HR on leaves of the nonhost pepper (*Capsicum annuum* cv. ECW-10R). In contrast, genetic complementation restored the virulence and ability to induce HR induction to levels comparable to the wild type.

**Fig 2:**
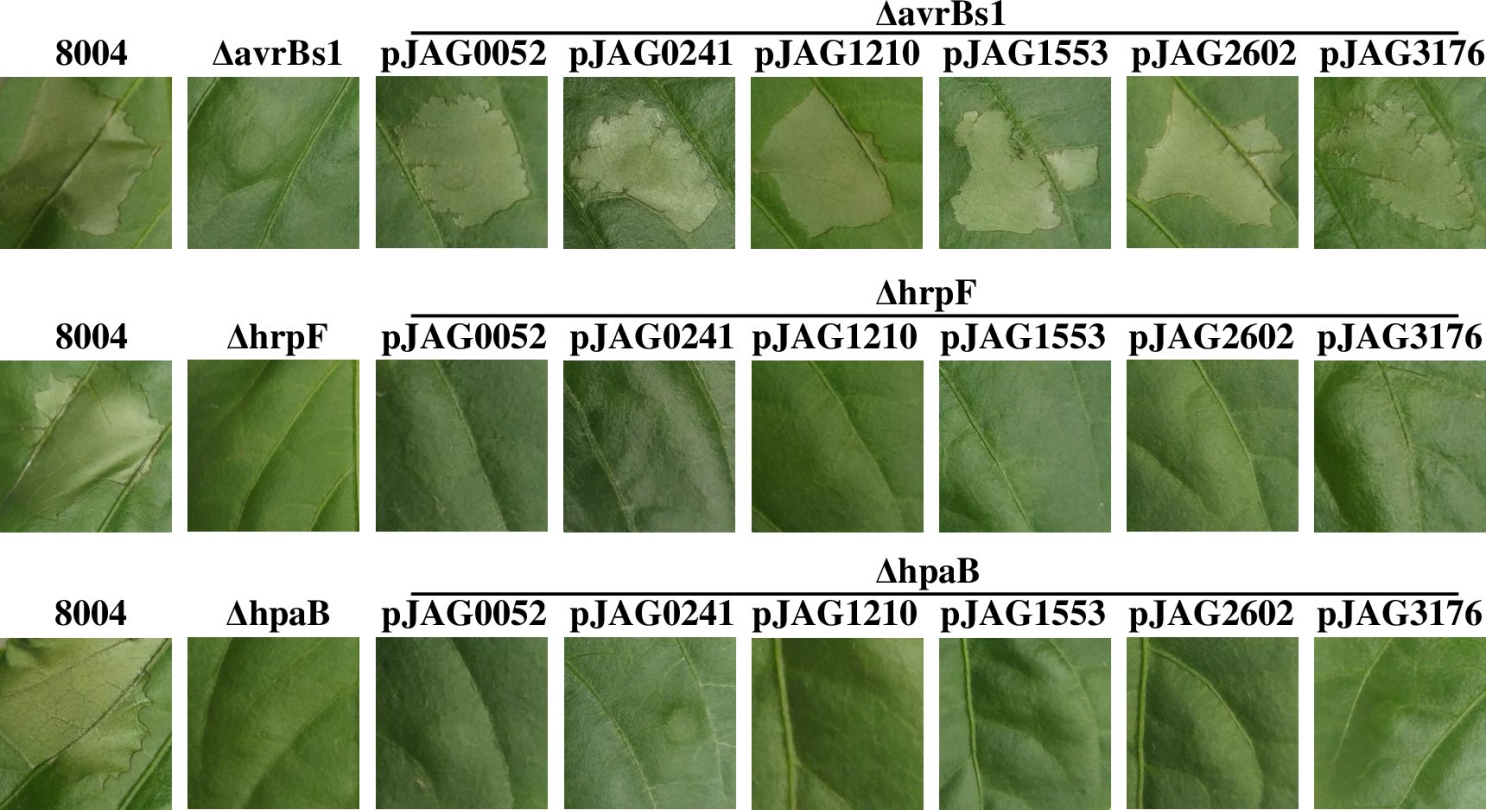
The translocation of T3Es is dependent on HpaB in *Xcc*. The mutants ΔavrBs1, ΔhpaB, and ΔhrpF carrying plasmids that respectively encode N-terminal signals of XC0052, XC0241, XC1210, XC1553, XC2602, and XC3176, in-frame fused with AvrBs1_59-445_−3×FLAG were infiltrated into the expanded leaves of pepper plant (*Capsicum annuum* cv. ECW-10R) at an OD_600_ of 0.1. The phenotypes were photographed at 24 h post-inoculation. The wild type strain 8004 was used here as the positive control and the deletion mutants ΔavrBs1 and ΔhrpF as the negative control.

*Xcc* carries at least thirteen T3Es with some variations between strains [18,35,41–45]. Previous work has identified that translocation of six of these T3Es (XC2994, XC2995, XC3160, XC3177, XC3802, and XC4273) depends on HpaB [18]. T3Es have a common, modular structure, with an N terminal secretion signal required for secretion and translocation through T3S machine [34,46]. To examine whether the translocation of any remaining T3Es (XC0052, XC0241, XC1210, XC1553, XC2602, and XC3176) also depends on HpaB, clones expressing fusions of their N-terminal regions (about 150 amino acids) with AvrBs1 (residues 59–445) were created for HR assays in *Capsicum annuum* cv. ECW-10R, which carries the *Bs1* resistance gene [18]. AvrBs1_59-445_ fails in translocation due to the lack of an N-terminal translocation signal. The resulting recombinant plasmids were introduced into ΔavrBs1, ΔhpaB, and ΔhrpF strains, respectively. The translocation assay in AvrBs1-responsive pepper leaves showed that all of these hybrid effectors could restore the ability to induce HR to the ΔavrBs1 strain. However, none of these hybrid effectors was capable of inducing HR in either the ΔhpaB or ΔhrpF strains, although the hybrid effectors were expressed by *Xcc* strains (Figs 2 and S2A). This data suggested that HpaB serves as chaperone function for translocation of this panel of tested effectors.

### The C-terminal domain of HpaB is involved in virulence and HR induction in *Xcc*

Sequence alignment analysis showed that HpaB shares more than 80% identity and 90% similarity among *Xanthomonas citri* pv. *citri* 306, *Xanthomonas oryzae* pv. *oryzae* KACC 10331, *Xanthomonas campestris* pv. *vesicatoria*, *Xanthomonas axonopodis* pv. *citrumelo* F1, and *Xanthomonas oryzae* pv. *oryzicola* BLS256, and over 45% identity and 60% similarity in *Xanthomonas translucens*, *Rhizobacter gummiphilus*, and *Ralstonia solanacearum* GMI1000. Furthermore, the C-terminal region of HpaB shares higher identity and similarity between those homologs (S3 Table). This result indicated that C-terminal region of HpaB potentially plays a crucial role for protein function.

To examine the function of the C-terminal region of HpaB, *Xcc* strains expressing truncated variants lacking either the C-terminal domain (amino acid residues 137–160) or part of the CesT domain (amino acid residues 45–160) of HpaB were generated and named Δ137–160 and Δ45–160, respectively (S1 Table). As with ΔhpaB both Δ137–160 and Δ45–160 failed to create disease symptoms on the host plant Chinese radish (*Raphanus sativus* var. *radiculus* cv. Manshenhong) and could not elicit an HR on nonhost pepper (*Capsicum annuum* cv. ECW-10R) leaves. The introduction of the full length *hpaB* cloned into pLAFRJ restored virulence and an ability to induce HR comparable to the wild type to both strains (Fig 1). These results suggested that the C-terminal domain plays important role for HpaB function in *Xcc*.

### The C-terminal domain of HpaB is required for the secretion and translocation of type III effectors in *Xcc*

The loss of plant phenotypes after truncation of the C-terminal domain of HpaB prompted us to ask whether the deletion mutant was affected in the secretion/translocation of T3Es. To address this conjecture, we analyzed the *in vitro* secretion of T3Es in different *hpaB* deletion derivatives by Western blotting and *in planta* translocation using fusion proteins with AvrBs1 and Cya.

**Fig 3:**
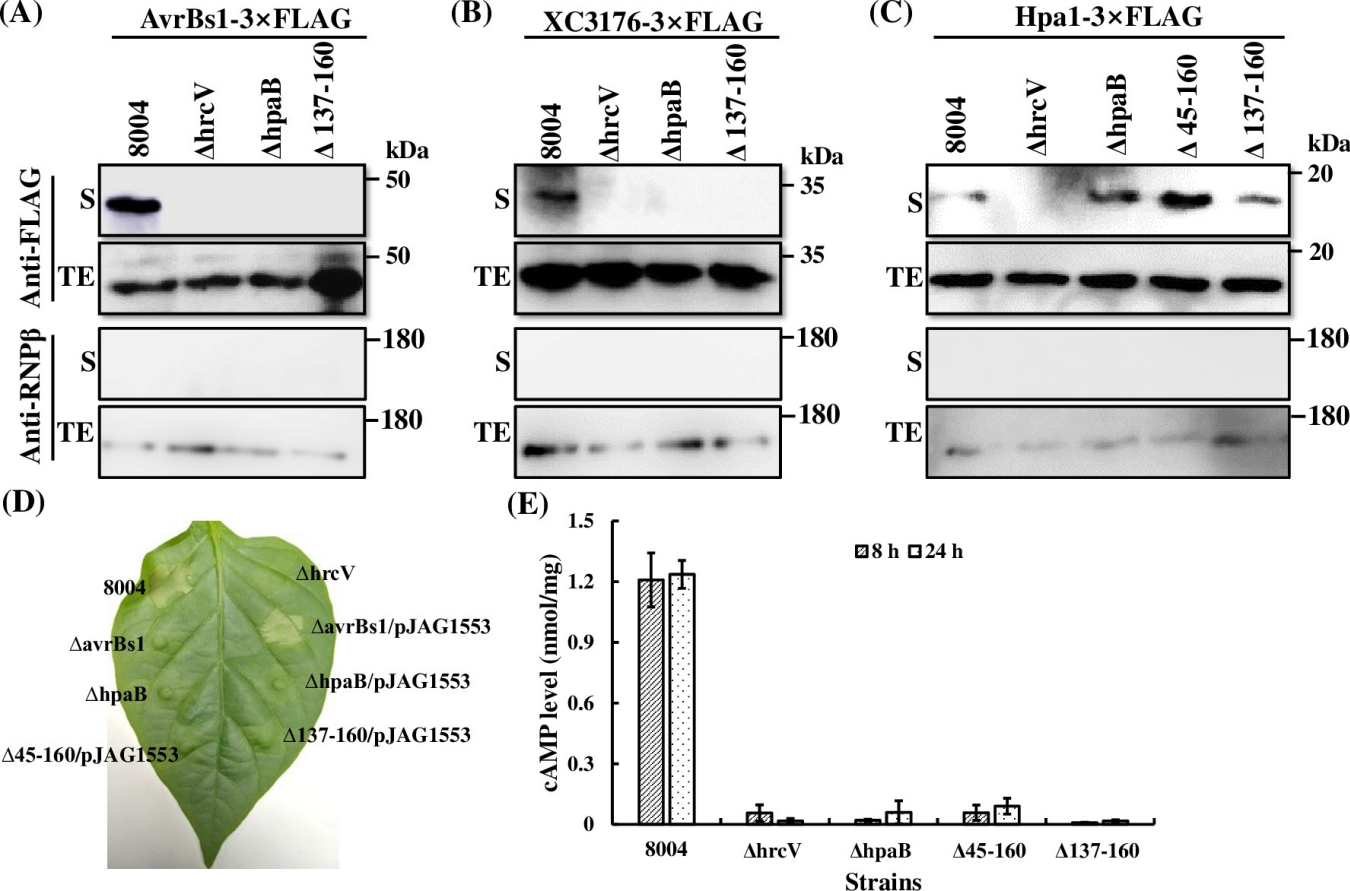
The C-terminal domain of HpaB is required for the secretion and translocation of T3Es in *Xcc*. (**A-C**) *Xcc* strains were incubated in *hrp*-inducing medium MMX. Total cell extracts (TE) and culture supernatants (S) were analyzed by immunoblotting with an ant-FLAG antibody or anti-RNP*β* antibody. T3Es AvrBs1 (**A**) and XC3176 (**B**) tagged with 3×FLAG epitope tag were introduced into wild type strain 8004, T3S-defective ΔhrcV, ΔhpaB, and *hpaB* C-terminal deletion mutant Δ137–160, respectively. (**C**) Non-effector Hpa1 tagged with 3×FLAG epitope tag was introduced to wild type strain 8004, T3S-defective ΔhrcV, ΔhpaB, Δ45–160, and *hpaB* C-terminal deletion mutant Δ137–160, respectively. (**D**) Translocation assays were performed based AvrBs1 reporter. *Xcc* strains harboring or not with plasmid pJAG1553 expressing AvrAC_1-102_-AvrBs1_59-445_ fusion were infiltrated into the expanded leaves of pepper (*Capsicum annuum* cv. ECW-10R) at an OD_600_ of 0.1, respectively. The phenotypes were photographed at 24 h post-inoculation. Wild type strain 8004 and ΔhpaB were used here as positive and negative control, respectively. (**E**) Translocation assays were performed with Cya reporter. Wild type strain 8004, T3S-defective ΔhrcV, ΔhpaB, Δ45–160, and Δ137–160, with plasmid pJAA1553 expressing AvrAC_1-102_-Cya_2-400_−3×FLAG fusion were infiltrated into Chinese cabbage (*Brassica oleracea* cv. Jingfeng NO. 1) leaves at an OD_600_ of 0.1. The cAMP level found in infected leaves was tested at 8 and 24 h post-inoculation, respectively. Values are the mean ± standard deviation from three repeats, each with 3 leaves.

For Western analysis, the ORFs of *avrBs1* and *XC3176* [31] were cloned into the plasmid pJXG that contains a synthetic DNA fragment encoding 3×FLAG [18]. This construct was introduced into the wild-type strain 8004, *hpaB* deletion mutant strain ΔhpaB, the *hpaB* deletion derivative Δ137–160, and the T3SS-defective *hrcV* mutant ΔhrcV. These transconjugant strains were first cultured overnight in NYG medium and then transferred to a *hrp*-inducing MMX medium (minimal medium for *Xanthomonas campestris*) [30] at a bacterial density of OD_600_ = 0.1 before incubation for 8 h. As expected, T3E proteins AvrBs1-3×FLAG and XC3176-3×FLAG were present in the cells of all the strains tested and the culture supernatant of the wild type strain (Fig 3A and 3B). However, AvrBs1-3×FLAG and XC3176-3×FLAG proteins were undetectable in the cultural supernatant of the *hpaB* deletion mutations under the test conditions (Fig 3A and 3B). No signal was seen in the culture supernatant of the T3SS-defective control strain ΔhrcV.

*In planta* translocation assays were done with the T3E AvrAC fused with AvrBs1_59-445_ or the calmodulin-dependent reporter protein Cya [33]. The *XC1553* gene encodes the T3E AvrAC that is recognized in vascular tissues [35] and displays uridylyl transferase activity targeting *Arabidopsis* BIK1 and RIPK [47]. An 894-bp DNA fragment comprising 588 bp upstream of the start codon and the first 102 codons of *avrAC* was cloned into pJAG [18], which encodes AvrBs1_59-445_ to produce pJAG1553. While ΔavrBs1/pJAG1553 could induce a significant HR, no visible HR was elicited by the transconjugant strains ΔhpaB/pJAG1553, Δ45-160/pJAG1553, and Δ137-160/pJAG1553 at 24 h after inoculation (Fig 3D). However, the AvrAC_1-102_-AvrBs1_59-445_ chimera was expressed by all of *Xcc* strains (S2B Fig). These results suggested that this hybrid effector protein could be translocated by the wild type but not by any of the *hpaB* deletion mutants.

A construct expressing the N-terminal 102 amino acids of the AvrAC fused with the calmodulin-dependent reporter protein Cya_2-400_ was also constructed and named pJAA1553 (S1 Table). This construct was introduced into the wild-type strain 8004, *hpaB* deletion mutant strain ΔhpaB, Δ137–160, Δ45–160, and the T3SS-defective *hrcV* mutant ΔhrcV. The AvrAC_1-102_-Cya_2-400_ chimera was expressed by *Xcc* strains (S2C Fig). And then the transconjugant strains were inoculated into Chinese cabbage (*Brassica oleracea* cv. Jingfeng NO. 1) leaves at an OD_600_ of 0.1, and the cAMP levels were measured at 8 h or 24 h post-inoculation. As shown in Fig 3E, the level of cAMP in cabbage leaves inoculated with *hpaB* mutant strain ΔhpaB, Δ137–160 and Δ45–160 carrying the plasmid, was far less than wild type and similar to that seen with the negative control ΔhrcV. Taken together, these results suggested that the C-terminal domain of HpaB is required for type III effector translocation.

In addition to effects on T3Es, we examined the effect of HpaB and truncated derivatives on secretion of Hpa1, a homolog of the *Xanthomonas* outer protein XopA that belongs to a class of non-effector that can be secreted in absence of HpaB [16]. To test the secretion of Hpa1 in different *hpaB* deletion mutants, Hpa1 was tagged with 3×FLAG as outlined above. No Hpa1-3×FLAG protein was detected in the culture supernatant of T3SS-negative strain ΔhrcV (Fig 3C). Surprisingly, however, the amounts of Hpa1-3×FLAG protein in the culture supernatant of both ΔhpaB and Δ45–160 were higher than in the wild type strain 8004, whereas the levels in Δ137–160 was similar to that of wild type strain 8004. These data suggested that the C-terminal domain of HpaB is required for the secretion of T3E proteins but not for Hpa1.

### The C-terminal domain of HpaB is dispensable for interaction with type III effectors and self-association in *Xcc*

It was previously reported for chaperones in animal pathogenic bacteria that the predicted amphiphilic C-terminal end is involved in effector binding, whereas the N-terminal region is implicated in chaperone dimerization [40]. To examine whether the C-terminal domain of HpaB contributes to chaperone-effector interaction, glutathione S-transferase (GST) pull-down assays were performed. For this, GST and GST-fused HpaB derivatives were expressed in *E*. *coli*, immobilized on MagneGST^TM^ particles, and then incubated with *E*. *coli* lysates containing 6×His-AvrBs1 and 6×His-HpaA, respectively. HpaA is a regulator of the T3SS which is known to interact with HpaB and is crucial for the secretion of the *hrp* pilus and translocon; HpaA is itself secreted and translocated into plant cells [22]. The interaction of HpaA and HpaB promotes secretion of non-effector proteins; secretion of HpaA is thought to liberate HpaB and hence activate effector secretion [22].

**Fig 4:**
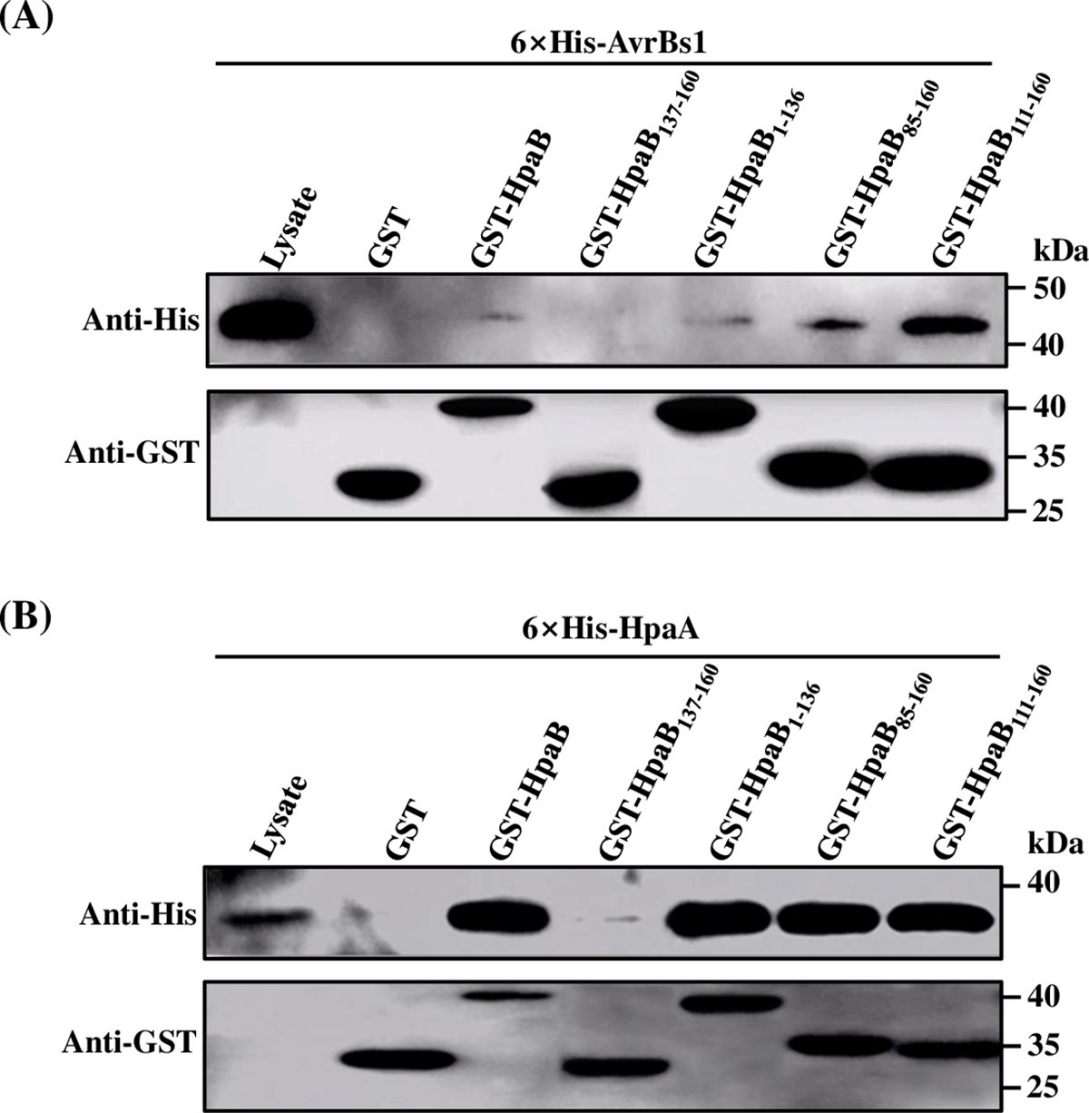
The C-terminal domain of HpaB is dispensable for interaction with T3Es in *Xcc*. (**A-B**) GST alone and GST-fused HpaB derivates were immobilized on MagneGST^TM^ particles and incubated with cell lysates containing 6×His-AvrBs1 (**A**) and 6×His-HpaA (**B**), respectively. Samples were analyzed using immunoblotting with hexahistidine-specific antibody and GST-specific antibody.

The findings showed that as seen with the negative control (GST alone) the C-terminal domain of HpaB (HpaB_137-160_) could not interact with AvrBs1 and only feebly interacted with HpaA (Fig 4). In contrast, both HpaB and its derivative lacking the C-terminal domain (HpaB_1-136_) could interact with AvrBs1 and HpaA (Fig 4). To confirm this, we examined the interactions between a further set of HpaB variants and effector proteins. Both 6×His-AvrBs1 and 6×His-HpaA were captured when incubated with GST-HpaB_85-160_, and GST-HpaB_111-160_ (Fig 4). Our data suggested that the site for interaction with effector proteins is likely located between amino acid residues 111 and 136, i.e., in the C-terminal region of the CesT domain of HpaB, which has a predicted amphiphilic helix.

**Fig 5:**
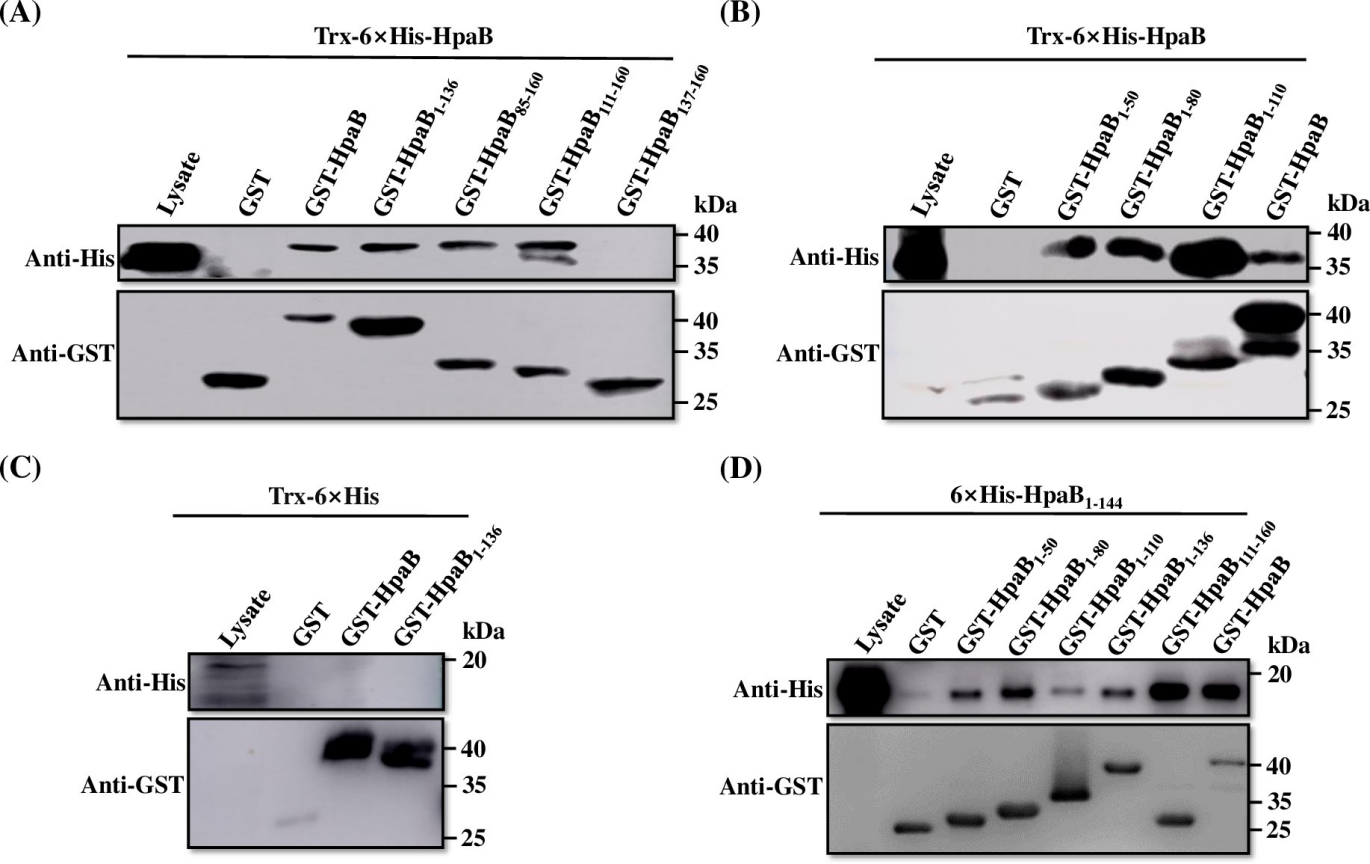
The C-terminal domain of HpaB is dispensable for self-interaction in *Xcc*. (**A-B**) GST and GST-fused HpaB derivates were immobilized on MagneGST^TM^ particles and incubated with cell lysate containing Trx-6×His-HpaB, respectively. Samples were analyzed by immunoblotting with hexahistidine-specific antibody and GST-specific antibody. (**C**) GST, GST-HpaB, and GST-HpaB_1-136_ were immobilized on MagneGST^TM^ particles and incubated with cell lysate containing Trx-6×His, as negative control. (**D**) GST and GST-fused HpaB derivates were respectively immobilized on MagneGST^TM^ particles and incubated with cell lysate containing 6×His-HpaB_1-144_.

Next, we determined whether HpaB derivatives could self-interact. All candidate interaction partners were immobilized as GST fusion proteins and incubated with bacterial lysates containing thioredoxin-fused HpaB (Trx-6×His-HpaB), respectively. As shown in Fig 5A and 5B, Trx-6×His-HpaB was captured by GST-HpaB, GST-HpaB_1-50_, GST-HpaB_1-80_, GST-HpaB_1-110_, GST-HpaB_1-136_, GST-HpaB_85-160_, and GST-HpaB_111-160_. As with GST alone, Trx-6×His-HpaB could not be detected in the elution product of GST-HpaB_137-160_ (Fig 5A). As a negative control, Trx-6×His could not be detected in the elution product of GST or GST fusion proteins (Fig 5C). To exclude the potential impact of Trx on HpaB protein function or protein-protein interaction, a construct expressing 6×His-HpaB_1-144_ was used in the same experiment and similar result was obtained (Fig 5D). Overall, these findings revealed that the C-terminal domain of HpaB has no effect on self-dimerization, suggesting that the C-terminal domain of HpaB (residues 137–160) may be dispensable for interactions with HpaB itself.

### The C-terminal domain of HpaB is dispensable for interaction with T3SS components in *Xcc*

**Fig 6:**
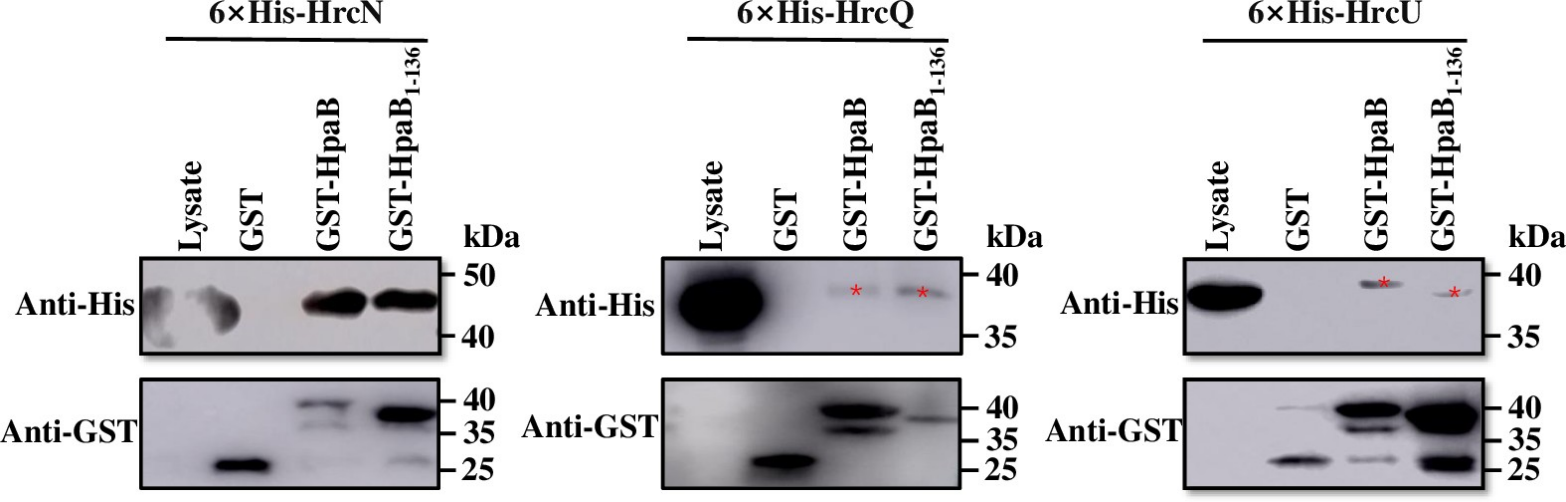
The C-terminal domain of HpaB is dispensable for interaction with T3SS components in *Xcc*. GST and GST-fused HpaB derivates were immobilized on MagneGST^TM^ particles and incubated with cell lysates containing 6×His-HrcN, 6×His-HrcQ, and 6×His-HrcU, respectively. Samples were analyzed using immunoblotting with hexahistidine-specific antibody and GST-specific antibody. The captured 6×His-HrcQ and 6×His-HrcU at weak level were remarked with asterisks.

Interestingly, a recent study has shown that many recently discovered T3Es target to bacterial membrane where chaperone HpaB promotes recognition of T3Es by T3SS [23]. The free HpaB targets to the cytoplasmic and/or inner components of T3SS, which presumably is used to regulate secretion and/or translocation of T3SS substrates [11,12,14,22,48,49]. Given that chaperone HpaB may contribute to effector targeting to T3SS, we examined possible interactions of HpaB and its C-terminally truncated variant with secretion platform components of T3SS, such as, HrcN, HrcQ, and HrcU using the GST-pulldown assays. As shown in Fig 6, HpaB could interact with HrcN, HrcQ, and HrcU, which is similar to what was observed with the same proteins in *Xcv* [11,12,22,49]. However, the C-terminally truncated HpaB could also bind to HrcN, HrcQ, and HrcU, suggesting that chaperone HpaB recognizes T3SS independent of its C-terminal domain (residues 137–160).

### HrcN, a T3SS-associated ATPase, is required for virulence and HR of *Xcc*

**Fig 7:**
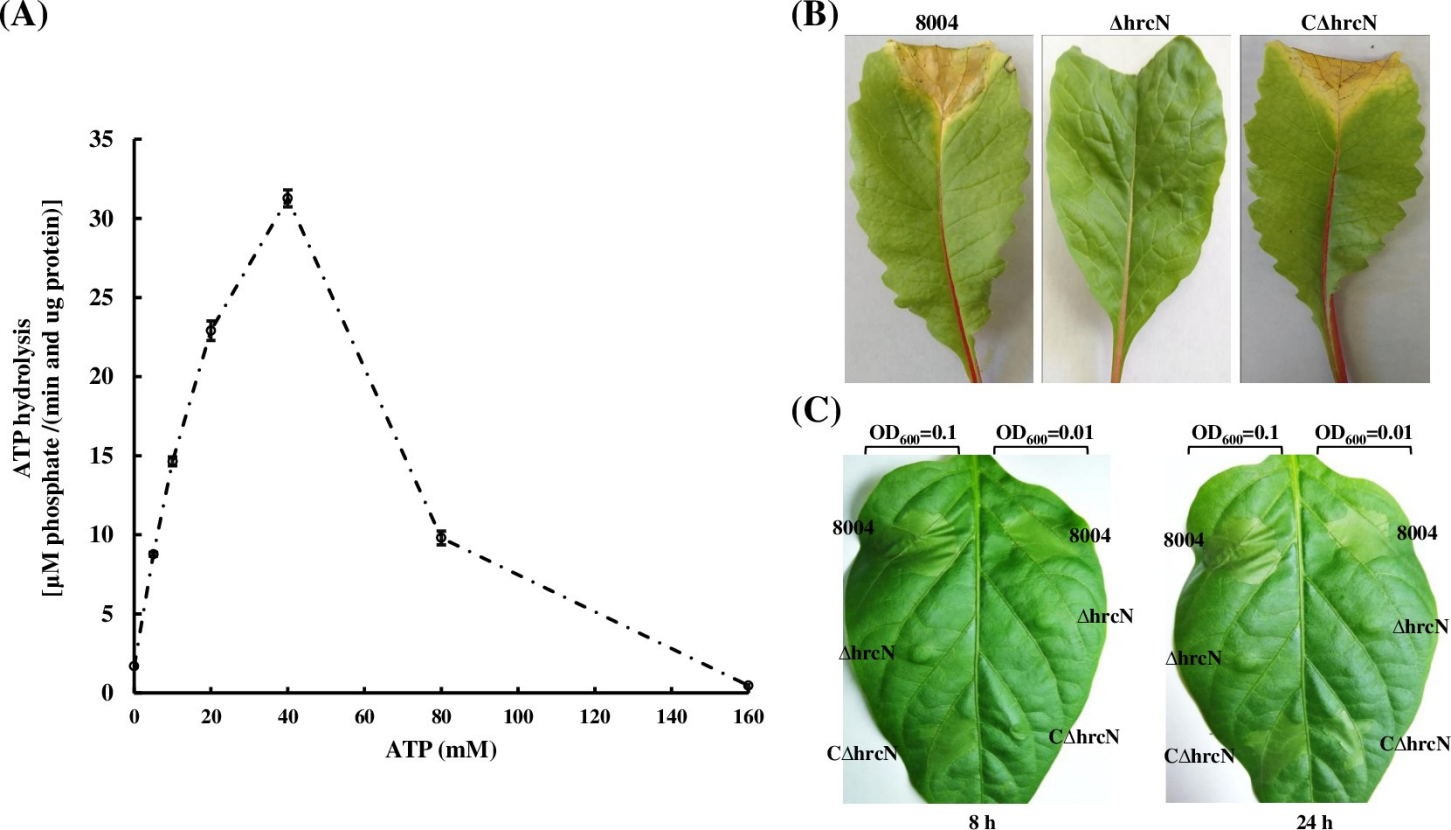
HrcN, a T3SS-associated ATPase, is required for virulence and HR of *Xcc*. (**A**) HrcN of *Xcc* exhibits ATP hydrolysis ability. ATP hydrolysis by 1 μg purified 6×His-HrcN was measured using a malachite green phosphatase assay in different concentrations of ATP. Values are the mean ± standard deviation from three repeats. (**B**) The lesion lengths of Chinese radish (*Raphanus sativus* var. *radiculus* cv. Manshenhong) leaves caused by *Xcc* strains (wild type strain 8004, *hrcN* deletion mutant ΔhrcN, and complementation of ΔhrcN CΔhrcN) 10 days post-inoculation. *Xcc* strains were diluted into 10 ml NYG medium at an OD_600_ of 0.001 and inoculated on Chinese radish by cutting-leaf method. (**C**) *Xcc* strains were inoculated into the leaves of pepper plant (*Capsicum annuum* cv. ECW-10R). Leaves were photographed at 8 h and 24 h post-inoculation respectively.

HrcN from *Xcc* is a highly conserved T3SS component, which shares more than 90% identity and 90% similarity in most of *Xanthomonas* spp., 43.0% identity and 60.0% similarity with EscN from *E*. *coli* O157:H7, and 41% identity and 57% similarity with InvC from *S*. *enterica*. HrcN and its homologues are posited at secretion platform of T3SS and function as a specific ATPase for energizing the T3SS [27,50–54]. In *Xcv*, HrcN is required for pathogenicity and T3S [11]. To explore the function of HrcN in *Xcc*, we first determined the effect of deletion of *hrcN* on bacterial ability in pathogenicity and HR-induction. As expected, the ΔhrcN strain had no virulence on host plant Chinese radish (*Raphanus sativus* var. *radiculus* cv. Manshenhong) and was not able to trigger HR on nonhost pepper (*Capsicum annuum* cv. ECW-10R); the genetic complementation (CΔhrcN) restored these plant phenotypes to wild type (Fig 7B and 7C). Overall, these results revealed that HrcN is involved in pathogenicity and HR in *Xcc*, where it probably participates in the secretion/translocation of effectors.

To determine whether HrcN of *Xcc* also has an ATPase activity, HrcN tagged with hexahistidine epitope tag was purified and the ATP hydrolysis activity under different concentrations of ATP was measured. As shown in Fig 7A, HrcN from *Xcc* exhibited an ATPase activity with optimal substrate concentration of 40 mM ATP; higher concentrations of ATP inhibited the ATPase activity. These results are consistent with the role of HrcN of *Xcv* as a T3SS-associated ATPase controlling T3S.

### The C-terminal domain of HpaB is required for ATPase-dependent dissociation of chaperone-effector complex

**Fig 8:**
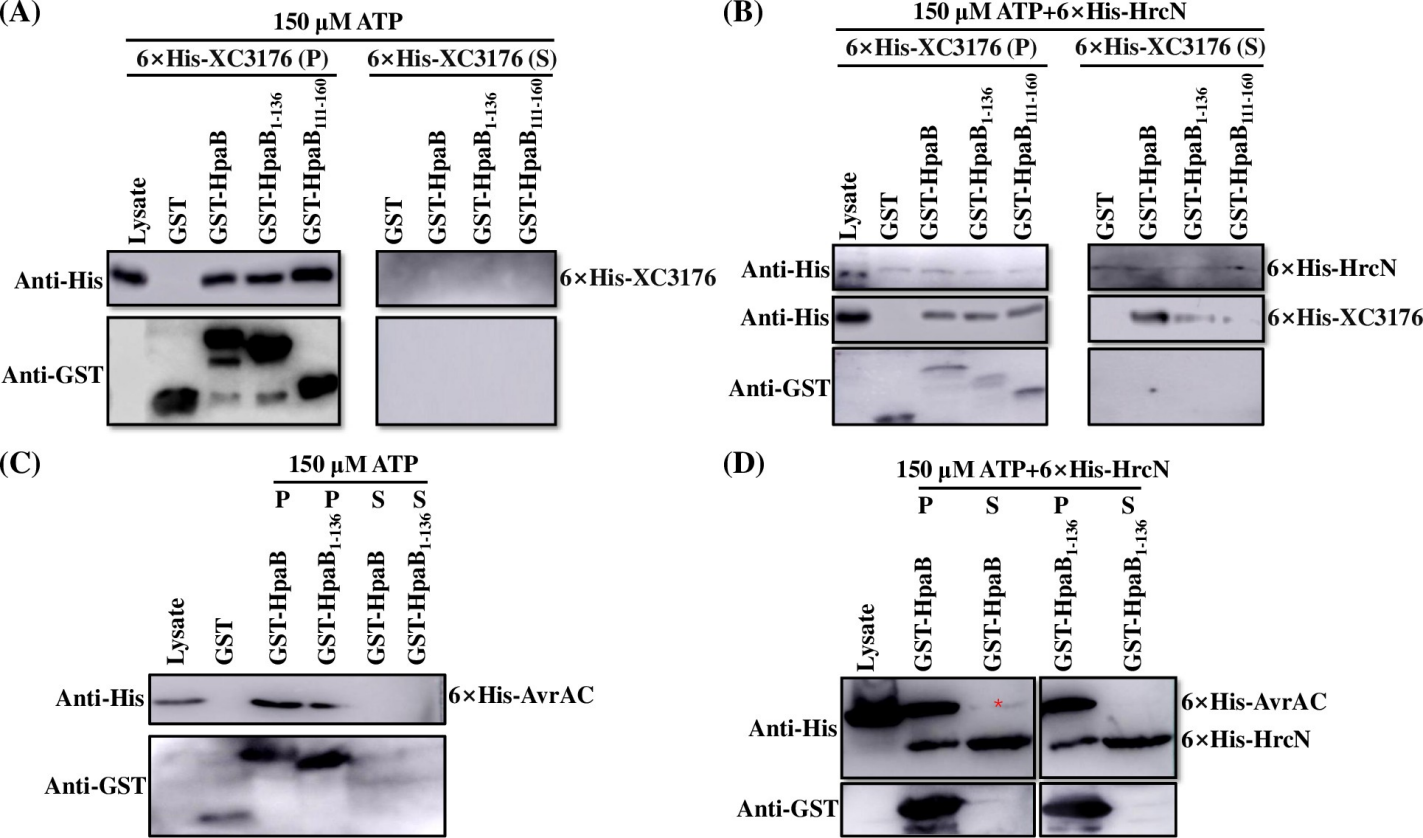
The C-terminal domain of HpaB is involved in ATPase-dependent dissociation of chaperone-effector complex in *Xcc*. (**A-B**) The T3E XC3176 binding to impaired chaperone is not released by purified 6×His-HrcN. Chaperone-effector complexes (GST-HpaB/6×His-XC3176, GST-HpaB_1-136_/6×His-XC3176, and GST-HpaB_111-160_/6×His-XC3176) and GST were absorbed on MagneGST^TM^ particles and incubated without (**A**) or with (**B**) 6×His-HrcN 1 h at room temperature in presence of 150 μM ATP, respectively. The released proteins in supernatant (S) and preyed proteins (P) on MagneGST^TM^ particles were separated by magnet. Samples were analyzed by immunoblotting with hexahistidine-specific antibody and GST-specific antibody. (**C-D**) The C-terminal domain of HpaB is crucial for liberating T3E AvrAC. (**C**) Chaperone-effector complexes of (GST-HpaB/6×His-AvrAC and GST-HpaB_1-136_/6×His-6×His-AvrAC and GST alone were absorbed on MagneGST^TM^ particles and incubated 1 h at room temperature in presence of 150 μM ATP, respectively. (**D**) One μg purified 6×His-HrcN was incubated with GST-HpaB/6×His-AvrAC and GST-HpaB_1-136_/6×His-AvrAC complex immobilized on MagneGST^TM^ particles, respectively, in presence of ATP. The released 6×His-AvrAC at a weak level was remarked with asterisk.

To better understand how the ATPase HrcN induces dissociation of chaperone-effector complex, we examined the effect of N-terminal and C-terminal truncations of HpaB on the dissociation of the complex of HpaB with the T3E XC3176. In initial experiments, the complexes of pre-bound wild-type GST-HpaB/6×His-XC3176, a C-terminally truncated GST-HpaB_1-136_/6×His-XC3176 and N-terminally truncated GST-HpaB_111-160_/6×His-XC3176 were incubated without ATPase at room temperature for 1 h in presence of 40 mM ATP (the optimal ATP concentration for HrcN) or 150 μM ATP [11] and the dissociation of the complexes were examined by Western blotting. The data (Figs 8A, 8C and S3) showed that effector XC3176 was partially released from complexes in presence of 40 mM ATP, but not 150 μM ATP, which suggested that the higher concentration of ATP could not be used in chaperone release assay. Interestingly, more 6×His-XC3176 was released from wild type GST-HpaB/6×His-XC3176 complex than from the truncated GST-HpaB/6×His-XC3176 complex in presence of 150 μM ATP and purified 6×His-HrcN (Fig 8B). Equivalent results were obtained when 6×His-AvrAC was incubated with GST-HpaB and GST-HpaB_1-136_ at same tested conditions (Fig 8C and 8D). These results reveal that the C-terminal domain of HpaB is required for efficient dissociation of itself from the chaperone-effector complex before effector secretion.

## Discussion

Interaction of chaperones with effectors leads to a partially unfolded or secretion-competent conformation of the effectors, which is essential for the secretion and translocation of effectors [24,40]. In this study, we have characterized some of the molecular details of the role of chaperone HpaB in secretion/translocation of effectors by the plant pathogen *Xcc*. Our findings indicate that the C-terminal domain of HpaB (the residues 137–160) is required for bacterial pathogenicity on a host plant and HR induction on a nonhost plant (Fig 1). These effects of deletion of the C-terminal domain are associated with an impairment in secretion and translocation of effectors (Fig 3), as seen both in *in vitro* and *in planta* tests. These findings are similar to what has been determined for the role of the HpaB homolog CesT in enteropathogenic *E*. *coli* (EPEC) [40,55]. Our data further suggest that the C-terminal domain of HpaB is not required for interaction with effector proteins nor (Figs 4 and 5).

Analysis of deletion derivatives indicates that a key site for interaction with effectors is probably located between amino acid residues 111 and 136, i.e., in the C-terminal region of the CesT domain of HpaB (Fig 4), which has a predicted amphiphilic helix. Multiple sites in HpaB appear to contribute to binding of effectors. This seems consistent with the conjecture [15] that a hydrophobic surface patch formed by the N-terminally amphipathic *α*1 and C-terminal *α*3 helices of T3S chaperones can make hydrophobic contact with effectors. Flexible modes of self-interaction of HpaB involving multiple sites may allow for different conformations that allow targeting of different substrates (Fig 5), including many of the T3Es and even some cytoplasmic platform components of T3SS in *Xcc* (Figs 4 and 6). We have shown here that HpaB is involved in secretion/translocation of six T3Es (Fig 2), in addition to the other six effectors previously described [18]. Although the secretion/translocation of almost all T3Es in *Xcc* is completely HpaB-dependent, in the closely related *Xcv*, some T3Es (class B), such as XopE2, XopB, and XopC, are still translocated in the absence of HpaB, but in significantly reduced amounts [48,56–58]. Most of these class B T3Es do not occur in *Xcc*, while a few T3Es have homologous proteins in *Xcc*, including XopK and XopE2 (encoded by *XC1210* and *XC2602* in *Xcc*, respectively), and whose translocations are completely dependent on HpaB. The reason for these differences in HpaB-dependency of different effectors is unclear.

The cytoplasmic domain of HrcV and the predicted C-ring component HrcQ provide docking sites for T3Es, which generally depend on a global chaperone HpaB or a substrate specificity switch (T3S4) HpaC to promote this process [12,49]. The cytoplasmic domain of HrcU can recognize early substrate HrpB2 but cannot bind to T3Es suggesting that HrcU may indirectly recognize T3Es [22,59]. The C ring components (HrcQ) and cytoplasmic domain of IM ring components (HrcU and HrcV) can also recognize chaperone HpaB [12,49]. We present here that HpaB can also bind to secretion platform components HrcN, HrcQ, and HrcU, and its C-terminal domain is dispensable for HpaB itself targeting to T3SS (Fig 6), which indicates the C-terminal deletion of HpaB may not affect the recognition of chaperone-effector complex by T3SS.

**Fig 9:**
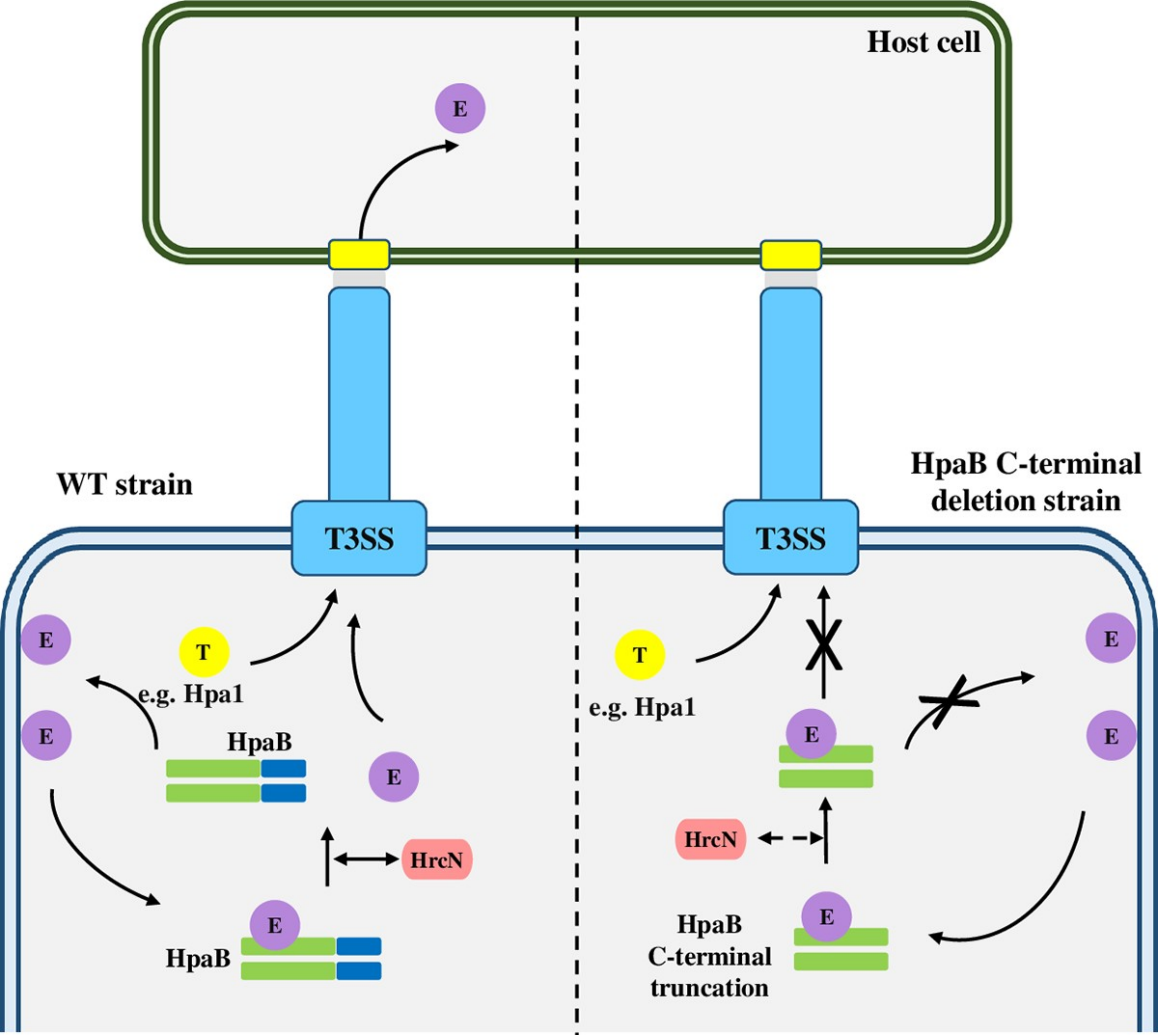
A working model for HpaB-dependent effector translocation in *Xcc*. In *Xcc* WT strain, HpaB binds to and promotes the secretion of various effector proteins (E) after the secretion of translocon proteins (T) via the T3SS. HpaB binding and secretion of effectors is facilitated by ATPase, HrcN. In the HpaB C-terminal deletion (right side), the truncated HpaB can bind various effector proteins (E) but appears to be unable to facilitate secretion. This indicates the C-terminal deletion of HpaB may not affect the recognition of chaperone-effector complex but may block secretion of effector proteins by forming a stable complex with these proteins, which blocks their dissociation and subsequent secretion of effectors.

T3Es must be released from chaperone-effector complexes before secretion and T3S-associated ATPases energize this progress to remove, unfold and recruit effector destined to the secretion platform [11,27]. We show that as in *Xcv*, HrcN of *Xcc* functions as an ATPase and is involved in bacterial pathogenicity and HR (Fig 7). Stimulation of this ATPase is probably associated with its oligomerization, the action of regulatory proteins such as HrcL, and Mg^2+^ [11,50–52,60–64]_._ The chaperone assay demonstrates that disassembly of effector proteins from chaperone-effector complexes relies on T3SS ATPase HrcN in *Xcc* (Fig 8). The C terminally truncated-HpaB may block secretion of effectors by forming a stable complex with effectors, which obstructs their dissociation and subsequent secretion of effectors (Figs 8 and 9).

## Supporting information

S1 Fig Sequence alignment of HpaB homologues. Homologous proteins were found using the BLASTP program (http://www.ncbi.nlm.nih.gov/blast/) in non-redundant databases based on matrix BLOSUM 62. Domain architectures of HpaB proteins were predicted on website (http://smart.embl-heidelberg.de/).
(PDF)

S2 Fig Immunoblotting analysis of *Xcc* strains. (**A**) *Xcc* strains (ΔavrBs1, ΔhrpF and ΔhpaB) carrying plasmids pJAG0052, pJAG0241, pJAG1210, pJAG1553, pJAG2602, and pJAG3176, respectively, were incubated in *hrp*-inducing medium MMX. Total cell extracts were analyzed by immunoblotting with ant-FLAG antibody. (**B-C**) *Xcc* strains carrying plasmids pJAG1553 (**B**) or pJAA1553 (**C**), respectively, were incubated in *hrp*-inducing medium MMX and were analyzed by immunoblotting with ant-FLAG antibody.
(PDF)

S3 Fig Chaperone release assay. Chaperone-effector complexes of (GST-HpaB/6×His-XC3176, GST-HpaB_1-136_/6×His-XC3176, and GST-HpaB_111-160_/6×His-XC3176) and GST were absorbed on MagneGST^TM^ particles and incubated without 6×His-HrcN 1 h at room temperature in presence of 40 mM ATP, respectively. The released proteins in supernatant (S) and preyed proteins (P) on MagneGST^TM^ particles were separated by magnet. Samples were analyzed by immunoblotting with hexahistidine-specific antibody and GST-specific antibody.
(PDF)

S1 Table Bacterial strains and plasmids used in this study. (DOCX)

S2 Table Oligo nucleotide sequences used in this study. (DOCX)

S3 Table Characteristics of HpaB homologue in phytopathogenic bacteria. (DOCX)

S1 Raw images (PDF)
