# Supplementary figures and images for: The C-terminal domain of the type III secretion chaperone HpaB contributes to dissociation of chaperone-effector complex in Xanthomonas campestris pv. campestris
Source: PLoS One. 2021 Jan 28;16(1):e0246033. doi: 10.1371/journal.pone.0246033 (PMC7842900; doi:10.1371/journal.pone.0246033)

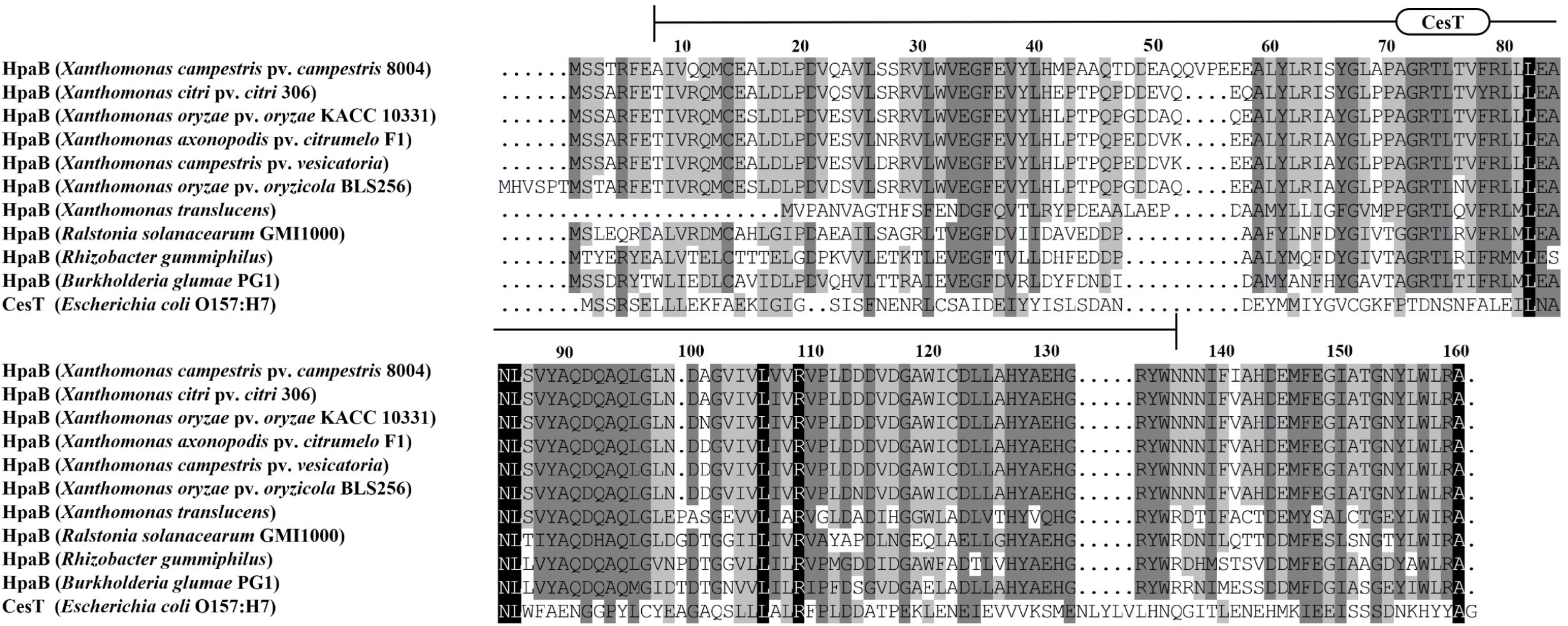

Supplement: S1 Fig — Homologous proteins were found using the BLASTP program (http://www.ncbi.nlm.nih.gov/blast/) in non-redundant databases based on matrix BLOSUM 62. Domain architectures of HpaB proteins were predicted on website (http://smart.embl-heidelberg.de/). (PDF) [file pone.0246033.s001.pdf]

(A)

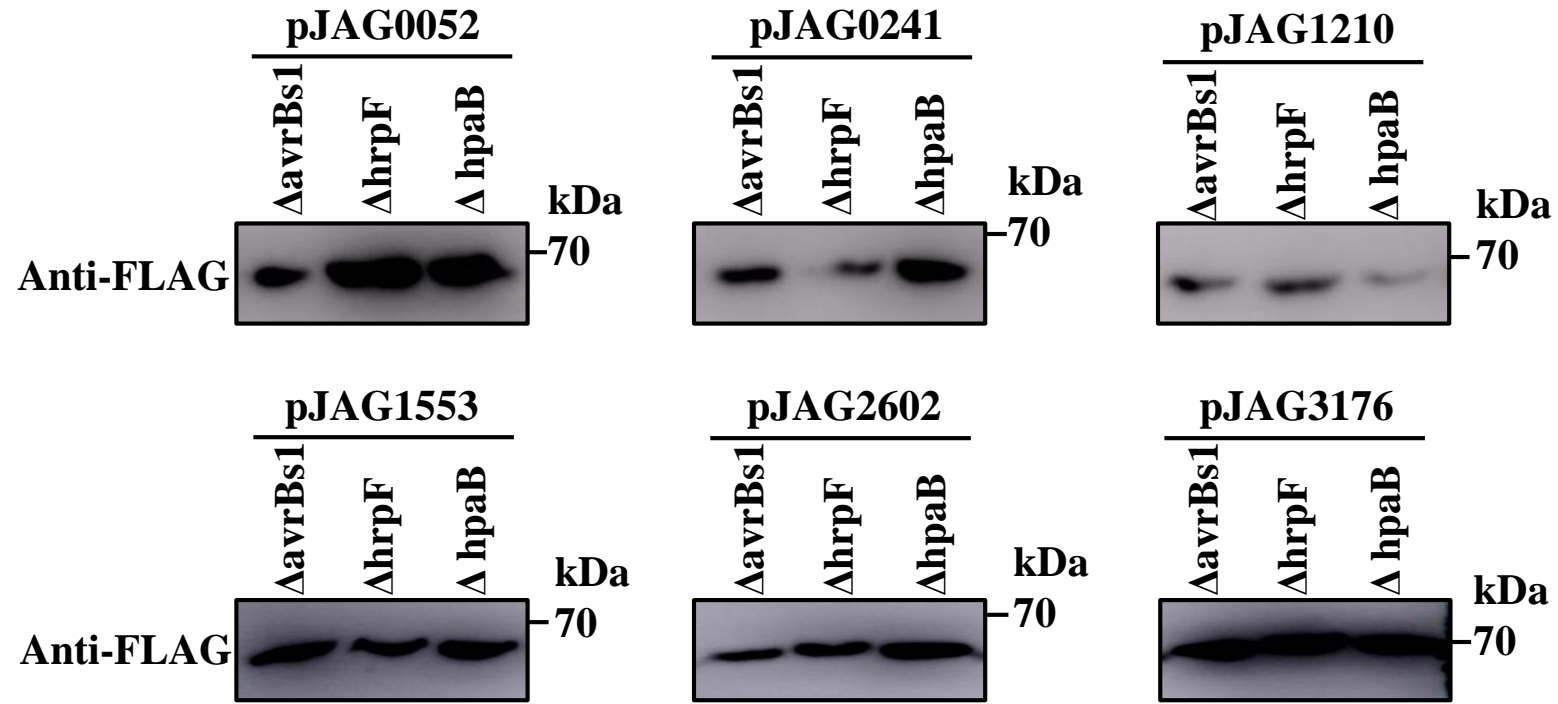

(B)

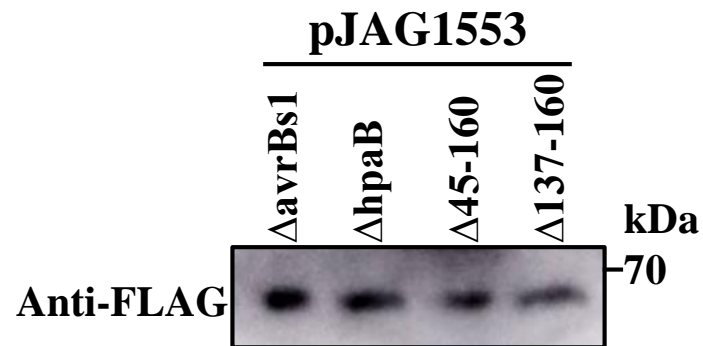

(C)

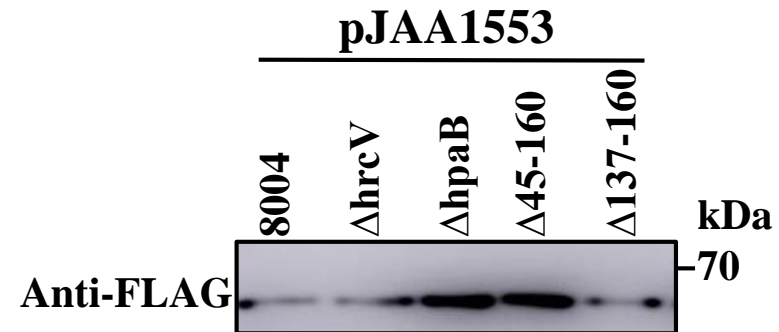

Supplement: S2 Fig — (A) Xcc strains (ΔavrBs1, ΔhrpF and ΔhpaB) carrying plasmids pJAG0052, pJAG0241, pJAG1210, pJAG1553, pJAG2602, and pJAG3176, respectively, were incubated in hrp-inducing medium MMX. Total cell extracts were analyzed by immunoblotting with ant-FLAG antibody. (B-C) Xcc strains carrying plasmids pJAG1553 (B) or pJAA1553 (C), respectively, were incubated in hrp-inducing medium MMX and were analyzed by immunoblotting with ant-FLAG antibody. (PDF) [file pone.0246033.s002.pdf]

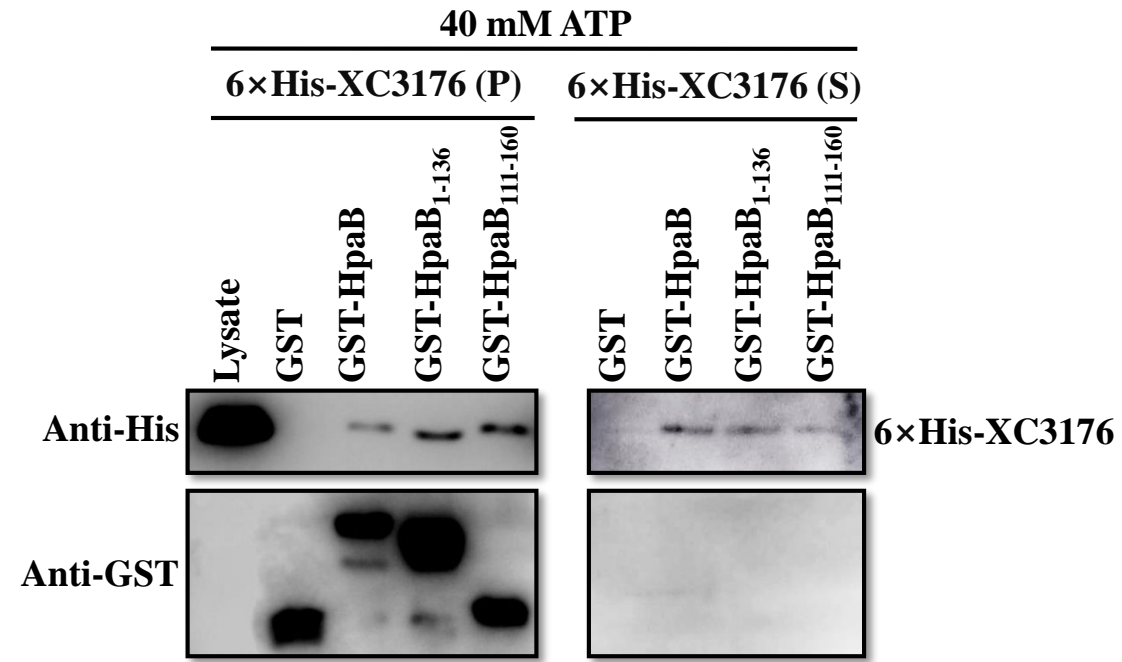

Supplement: S3 Fig — Chaperone-effector complexes of (GST-HpaB/6×His-XC3176, GST-HpaB1-136/6×His-XC3176, and GST-HpaB111-160/6×His-XC3176) and GST were absorbed on MagneGSTTM particles and incubated without 6×His-HrcN 1 h at room temperature in presence of 40 mM ATP, respectively. The released proteins in supernatant (S) and preyed proteins (P) on MagneGSTTM particles were separated by magnet. Samples were analyzed by immunoblotting with hexahistidine-specific antibody and GST-specific antibody. (PDF) [file pone.0246033.s003.pdf]
