# Supplementary material for: The C-terminal domain of the type III secretion chaperone HpaB contributes to dissociation of chaperone-effector complex in Xanthomonas campestris pv. campestris
Source: PLoS One. 2021 Jan 28;16(1):e0246033. doi: 10.1371/journal.pone.0246033 (PMC7842900; doi:10.1371/journal.pone.0246033)
Supplement: S1 Table — (DOCX) [file pone.0246033.s004.docx]

**Table S1 Bacterial strains and plasmids used in this study**

| **Names** | **Characteristics** | **References or sources** |
| --- | --- | --- |
| *Xanthomonas campestris* pv. *campestris* strains | | |
| 8004 | Wild type, Rif^r^ | [1] |
| ΔavrBs1 | deletion of *avrBs1* gene in 8004, Rif^r^ | Our lab's collection |
| ΔhrcV | deletion of *hrcV* gene in 8004, Rif^r^ | Our lab's collection |
| ΔhrcN | deletion of *hrcN* gene in 8004, Rif^r^ | Our lab's collection |
| ΔhrpF | deletion of *hrpF* gene in 8004, Rif^r^ | Our lab's collection |
| ΔhpaB | in frame deletion of *hpaB* gene in 8004, Rif^r^ | This study |
| Δ45-160 | in frame deletion of 45-160 codons of *hpaB* gene in 8004, Rif^r^ | This study |
| Δ137-160 | in frame deletion of 137-160 codons of *hpaB* gene in 8004, Rif^r^ | This study |
| CΔhrcN | complementation of ΔhrcN containing pJNhrcN, Rif^r^, Tc^r^ | This study |
| CΔhpaB | complementation of ΔhpaB containing pJNhpaB, Rif^r^, Tc^r^ | This study |
| CΔ45-160 | complementation of Δ45-160 containing pJNhpaB, Rif^r^, Tc^r^ | This study |
| CΔ137-160 | complementation of Δ137-160 containing pJNhpaB, Rif^r^, Tc^r^ | This study |
| ΔavrBs1/pJAG1553 | ΔavrBs1 containing pJAG1553, Rif^r^, Tc^r^ | This study |
| ΔhpaB/pJAG1553 | ΔhpaB containing pJAG1553, Rif^r^, Tc^r^ | This study |
| Δ45-160/pJAG1553 | Δ45-160 containing pJAG1553, Rif^r^, Tc^r^ | This study |
| Δ137-160/pJAG1553 | Δ137-160 containing pJAG1553, Rif^r^, Tc^r^ | This study |
|  |  |  |
| *E*. *coli* | | |
| DH5*α* | F^-^ *recA* *hsdR17* (*rk^-^*, *mk^+^*) *𝛷80* *dLacZ* *DM15* | Gibco BRL, Life Technologies |
| BL21(DE3) | F^-^ *ompT* *hsdR17* (*rB^-^*, *mB^+^*) *gal* *dcm* (*DE3*) | Novagen |
| C43(DE3) | derivative of BL21(DE3), better expression of toxic proteins | [2] |
| ED8767/pRK2073 | Helper strain containing pRK2073, *recA met*, Spc^r^ | [3] |
|  |  |  |
| plasmids |  |  |
| pK18mobsacB | Suicide plasmid, *sacB*, Kan^r^ | [4] |
| pK18-ΔhpaB | derivative of pK18mobsacB containing hpaB flanking regions, Kan^r^ | This study |
| pK18-Δ45-160 | derivative of pK18mobsacB containing flanking-regions of 45-160 codons of *hpaB*, Kan^r^ | This study |
| pK18-Δ137-160 | derivative of pK18mobsacB containing flanking-regions of 137-160 codons of *hpaB*, Kan^r^ | This study |
| pET30a | Expression plasmid, T7 promoter, Kan^r^ | Novagen |
| pET30a-1553 | derivative of pET30a encoding 6×His-AvrAC, Kan^r^ | This study |
| pET30a-2081 | derivative of pET30a encoding 6×His-AvrBs1, Kan^r^ | This study |
| pET30a-3006 | derivative of pET30a encoding 6×His-HrcN, Kan^r^ | Our lab's collection |
| pET30a-3012 | derivative of pET30a encoding 6×His-HrcU, Kan^r^ | Our lab's collection |
| pET30a-3015 | derivative of pET30a encoding 6×His-HrcQ, Kan^r^ | Our lab's collection |
| pET30a-3018 | derivative of pET30a encoding 6×His-HpaA, Kan^r^ | Our lab's collection |
| pET30a-HpaB_1-144_ | derivative of pET30a encoding 6×His-HpaB_1-144_, Kan^r^ | This study |
| pET30a-3176 | derivative of pET30a encoding 6×His-XC3176, Kan^r^ | This study |
| pET32a | Expression plasmid, T7 promoter, N-Trx tag, Amp^r^ | Novagen |
| pET32a-HpaB | derivative of pET32a encoding Trx-6×His-HpaB, Amp^r^ | This study |
| pGEX-4T-1 | *gst* expression plasmid, *Tac* promoter, GST, pBR322 *ori*, Amp^r^ | Our lab's collection |
| pGEXHpaB | pGEX-4T-1 derivative encoding GST-HpaB, Amp^r^ | This study |
| pGEXHpaB_1-50_ | pGEX-4T-1 derivative encoding GST-HpaB_1-50_, Amp^r^ | This study |
| pGEXHpaB_1-80_ | pGEX-4T-1 derivative encoding GST-HpaB_1-80_, Amp^r^ | This study |
| pGEXHpaB_1-110_ | pGEX-4T-1 derivative encoding GST-HpaB_1-110_, Amp^r^ | This study |
| pGEXHpaB_1-136_ | pGEX-4T-1 derivative encoding GST-HpaB_1-136_, Amp^r^ | This study |
| pGEXHpaB_85-160_ | pGEX-4T-1 derivative encoding GST-HpaB_85-160_, Amp^r^ | This study |
| pGEXHpaB_111-160_ | pGEX-4T-1 derivative encoding GST-HpaB_111-160_, Amp^r^ | This study |
| pGEXHpaB_137-160_ | pGEX-4T-1 derivative encoding GST-HpaB_137-160_, Amp^r^ | This study |
| pLAFRJ | Broad host range plasmid, pLAFR3 derivative containing the multiple cloning sites of pUC19, Tc^r^ | [5] |
| pJXG | Broad host range plasmid, pLAFRJ derivative containing DNA fragment encoding 3×FLAG, Tc^r^ | [5] |
| pJAG | Broad host range plasmid, pJXG derivative containing DNA fragment encoding AvrBs1_59-445_, Tc^r^ | [5] |
| pJAA | Broad host range plasmid, pJXG derivative containing DNA fragment encoding Cya, Tc^r^ | [6] |
| pJNhpaB | pLAFRJ derivative containing DNA fragment of 500 bp upstream, ORF and 100 bp downstream of *hpaB*, Tc^r^ | This study |
| pJNhrcN | pLAFRJ derivative containing DNA fragment of 498 bp upstream, ORF and 93 bp downstream of *hrcN*, Tc^r^ | Our lab's collection |
| pJAG0052 | pJAG derivative containing DNA fragment of 555 bp upstream and first 161 codons of *XC0052* (*avrBs2*), Tc^r^ | Our lab's collection |
| pJAG0241 | pJAG derivative containing DNA fragment of 488 bp upstream and first 53 codons of *XC0241* (*xopXccN*), Tc^r^ | Our lab's collection |
| pJAG1210 | pJAG derivative containing DNA fragment of 705 bp upstream and first 131 codons of *XC1210* *(xopK*), Tc^r^ | Our lab's collection |
| pJAG1553 | pJAG derivative containing DNA fragment of 588 bp upstream and first 102 codons of *XC1553* (*avrAC*), Tc^r^ | Our lab's collection |
| pJAG2602 | pJAG derivative containing DNA fragment of 194 bp upstream and first 134 codons of *XC2602* (*avrXccE1*), Tc^r^ | Our lab's collection |
| pJAG3176 | pJAG derivative containing ORF of *XC3176*, Tc^r^ | This study |
| pJAA1553 | pJAA derivative containing DNA fragment of 588 bp upstream and first 102 codons of *XC1553* (*avrAC*), Tc^r^ | [6] |
| pJXG2081 | pJXG derivative containing ORF of *XC2081* (*avrBs1*), Tc^r^ | This study |
| pJXG3002 | pJXG derivative containing ORF of *XC3002* (*hpa1*), Tc^r^ | This study |
| pJXG3176 | pJXG derivative containing ORF of *XC3176*, Tc^r^ | [7] |

**References:**

1. Daniels MJ, Barber CE, Turner PC, Sawczyc MK, Byrde RJ, Fielding AH. Cloning of genes involved in pathogenicity of *Xanthomonas campestris* pv. *campestris* using the broad host range cosmid pLAFR1. EMBO J. 1984; 3(13):3323-8. <https://doi.org/10.1002/j.1460-2075.1984.tb02298.x> PMID: 16453595
2. Miroux B, Walker JE. Over-production of proteins in *Escherichia coli*: mutant hosts that allow synthesis of some membrane proteins and globular proteins at high levels. J Mol Biol. 1996; 260(3):289-98. <https://doi.org/10.1006/jmbi.1996.0399> PMID: 8757792.
3. Leong SA, Ditta GS, Helinski DR. Heme biosynthesis in *Rhizobium*. Identification of a cloned gene coding for delta-aminolevulinic acid synthetase from *Rhizobium meliloti*. J Biol Chem. 1982; 257(15):8724-30. <https://www.jbc.org/content/257/15/8724.long> PMID: 7096330.
4. Schäfer A, Tauch A, Jäger W, Kalinowski J, Thierbach G, Pühler A. Small mobilizable multi-purpose cloning vectors derived from the *Escherichia coli* plasmids pK18 and pK19: selection of defined deletions in the chromosome of *Corynebacterium glutamicum*. Gene. 1994; 145(1):69-73. <https://doi.org/10.1016/0378-1119(94)90324-7> PMID: 8045426.
5. Jiang W, Jiang BL, Xu RQ, Huang JD, Wei HY, Jiang GF, et al. Identification of six type III effector genes with the PIP box in *Xanthomonas campestris* pv. *campestris* and five of them contribute individually to full pathogenicity. Mol Plant Microbe Interact. 2009; 22(11):1401-11. <https://doi.org/10.1094/MPMI-22-11-1401> PMID: 19810809
6. Wang L, Yang LY, Gan YL, Yang F, Liang XL, Li WL, et al. Two lytic transglycosylases of *Xanthomonas campestris* pv. *campestris* associated with cell separation and type III secretion system, respectively. FEMS Microbiol Lett. 2019; 366(7):fnz073. <https://doi.org/10.1093/femsle/fnz073> PMID: 30977795
7. Yang LC, Gan YL, Yang LY, Jiang BL, Tang JL. Peptidoglycan hydrolysis mediated by the amidase AmiC and its LytM activator NlpD is critical for cell separation and virulence in the phytopathogen *Xanthomonas campestris*. Mol Plant Pathol. 2018; 19(7):1705-18. <https://doi.org/10.1111/mpp.12653> PMID: 29240286
