# Supplementary material for: The C-terminal domain of the type III secretion chaperone HpaB contributes to dissociation of chaperone-effector complex in Xanthomonas campestris pv. campestris
Source: PLoS One. 2021 Jan 28;16(1):e0246033. doi: 10.1371/journal.pone.0246033 (PMC7842900; doi:10.1371/journal.pone.0246033)
Supplement: S2 Table — (DOCX) [file pone.0246033.s005.docx]

**Table S2 Oligo nucleotide sequences used in this study**

| **Primers** | **Sequences** | **Purpose** |
| --- | --- | --- |
| DXC3022LFB | CCCGGATCCCTTGCAGACCAAGGCGCAGGATCTCTC | construction of ΔhpaB |
| DXC3022LRX | GGGTCTAGACTGTCGTACGACATGCGCAGGGC |  |
| DXC3022RFX | CCCTCTAGAGGTGCTCGGTCTTGCCTGCC |  |
| DXC3022RRH | CCCAAGCTTTGGTCTTGTTCTGGAGGCCG |  |
| DXC3022_45-160_LRX | CCCTCTAGAGGCTGCAGGCATGTGCAGATAGACTTCA | construction of Δ45-160 |
| DXC3022_137-160_LRX | CCCTCTAGAAACAACATCTTCATTGCACACGAC | construction of Δ137-160 |
| E3022FB | TTGGGATCCATGAGCAGCACCCGATTCGA | construction of 6×His-HpaB, Trx-6×His-HpaB, or GST-HpaB |
| E3022RE | GGGGAATTCCGCGCGTAGCCACAGATAAT |  |
| E3022FE | GGGGAATTCAGCAGCACCCGATTCGAAGC | construction of 6×His-HpaB, Trx-6×His-HpaB, or GST-HpaB |
| E3022RXho1 | TTGCTCGAGTCACGCGCGTAGCCACAGAT |  |
| E3022_1-50_RXh | GGGCTCGAGTTCATCGTCGGTCTGGGCTG | construction of GST-HpaB_1-50_ |
| E3022_1-80_RXh | TTGCTCGAGCAGCCGGAAGACCGTCAAGG | construction of GST-HpaB_1-80_ |
| E3022_1-110_RE | TTGGAATTCAACACGTACGACCAGCACGA | construction of GST-HpaB_1-110_ |
| E3022_1-136_RXh | TTGCTCGAGGTTCCAGTAACGGCCATGCT | construction of GST-HpaB_1-136_ |
| E3022_1-144_RE | GGGGAATTCGTGTGCAATGAAGATGTTGT | construction of 6×His-HpaB_1-144_ |
| E3022_85-160_FE | GGGGAATTCAATTTGTCGGTGTACGCACAGGACC | construction of GST-HpaB_85-160_ |
| E3022_111-160_FE | GGGGAATTCCCACTGGACGACGACGTCGA | construction of GST-HpaB_111-160_ |
| E3022_137-160+700_FE | GGGGAATTCAACAACATCTTCATTGCACACGAC | construction of GST-HpaB_137-160_ |
| E3022_137-160+700_RXh | TTGCTCGAGCGATGTAGGTCTGACCCTTG |  |
| E2081-FE | GGGGAATTCATGTCCGACATGAAAGTTAA | construction of 6×His-AvrBs1 |
| E2081-RXh | GGGCTCGAGTTACGCTTCTCCTGCATTTG |  |
| E3176-FE | GGGGAATTCGTGACTTCTGTGGCTCGTGA | construction of 6×His-XC3176 |
| E3176-RXh | GGGCTCGAGTCAACCATCGGACGATGCAG |  |
| E1553-FE | GGGGAATTCATGGATAAAAATCTTAATTTG | construction of 6×His-AvrAC |
| E1553-RXh | GGGCTCGAGCTACTGGTGAACCTGGTTCA |  |
| pLAFRJ3022FE | GGGGAATTCCAGGCTGCGCGCAACGATTT | construction of pJNhpaB |
| pLAFRJ3022RB | GGGGGATCCTGTCCTACAGCACAGCGGAG |  |
| pJXG2081FE | GGGGGAATTCAATGTCCGACATGAAAGTTAA | construction of pJXG2081 |
| pJXG2081RB | GGGGGATCCCGCTTCTCCTGCATTTGTAA |  |
| pJXG3002FE | GGGGGAATTCGGACTCATCTATCGGAAACA | construction of pJXG3002 |
| pJXG3002RB | AAAGGATCCTTGGCCGGTGATGCTCGACA |  |
| pJAG3176FE | GGGGAATTCAGTGACTTCTGTGGCTCGTGA | construction of pJAG3176 |
| pJAG3176RB | TTTGGATCCACCATCGGACGATGCAGACG |  |
