# Supplementary material for: The C-terminal domain of the type III secretion chaperone HpaB contributes to dissociation of chaperone-effector complex in Xanthomonas campestris pv. campestris
Source: PLoS One. 2021 Jan 28;16(1):e0246033. doi: 10.1371/journal.pone.0246033 (PMC7842900; doi:10.1371/journal.pone.0246033)
Supplement: S3 Table — (DOCX) [file pone.0246033.s006.docx]

**Table S3 Characteristics of HpaB homologue in phytopathogenic bacteria**

| **Bacteria** | **HpaB homolog**  **identity (%) / similarity (%)^a^** | **The C-terminal domain**  **identity (%) / similarity (%)^b^** |
| --- | --- | --- |
| *Xanthomonas campestris* pv*. campestris* 8004 | 100/100 | 100/100 |
| *Xanthomonas citri* pv*. citri* 306 | 88.1/91.9 | 95.8/100 |
| *Xanthomonas oryzae* pv. *oryzae* KACC 10331 | 86.3/91.9 | 95.8/100 |
| *Xanthomonas campestris* pv. *vesicatoria* | 85/91.3 | 95.8/100 |
| *Xanthomonas axonopodis* pv. *citrumelo* F1 | 85/91.9 | 95.8/100 |
| *Xanthomonas oryzae* pv*. oryzicola* BLS256 | 84.3/90.6 | 95.8/100 |
| *Xanthomonas translucens* | 47.2/62.1 | 58.8/70.6 |
| *Rhizobacter gummiphilus* | 50.9/69.6 | 76.5/88.2 |
| *Ralstonia solanacearum* GMI1000 | 50.3/66.5 | 58.8/64.7 |
| *Burkholderia glumae* PG1 | 53.4/68.9 | 65.2/69.6 |

^a^Homologous proteins found using the BLASTP program (http://www.ncbi.nlm.nih.gov/blast/) in non-redundant databases based on matrix BLOSUM 62.

^b^The C-terminal domain indicates 137-160 amino acid residues of HpaB from *Xanthomonas campestris* pv*. campestris* 8004.
