# Supplementary material for: The C-terminal domain of the type III secretion chaperone HpaB contributes to dissociation of chaperone-effector complex in Xanthomonas campestris pv. campestris
Source: PLoS One. 2021 Jan 28;16(1):e0246033. doi: 10.1371/journal.pone.0246033 (PMC7842900; doi:10.1371/journal.pone.0246033)
Supplement: S1 Raw images — (PDF) [file pone.0246033.s007.pdf]

Figure 3A-C

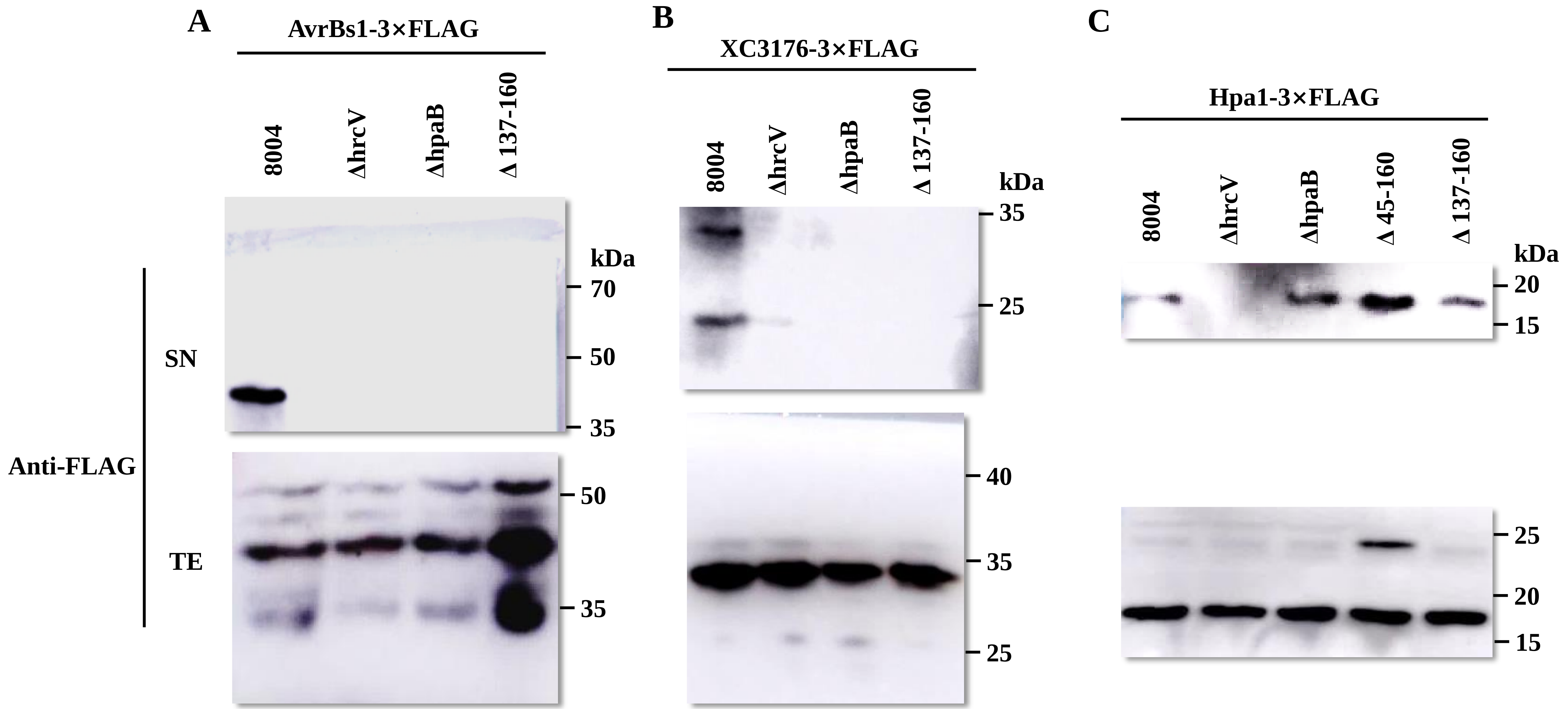
A
AvrBs1-3×FLAG
8004
ΔhrcV
ΔhpaB
Δ 137-160
kDa
70
50
35
SN
Anti-FLAG
TE
50
35
B
XC3176-3×FLAG
8004
ΔhrcV
ΔhpaB
Δ 137-160
kDa
35
25
40
35
25
C
Hpa1-3×FLAG
8004
ΔhrcV
ΔhpaB
Δ 45-160
Δ 137-160
kDa
20
15
25
20
15

Figure 3A-C

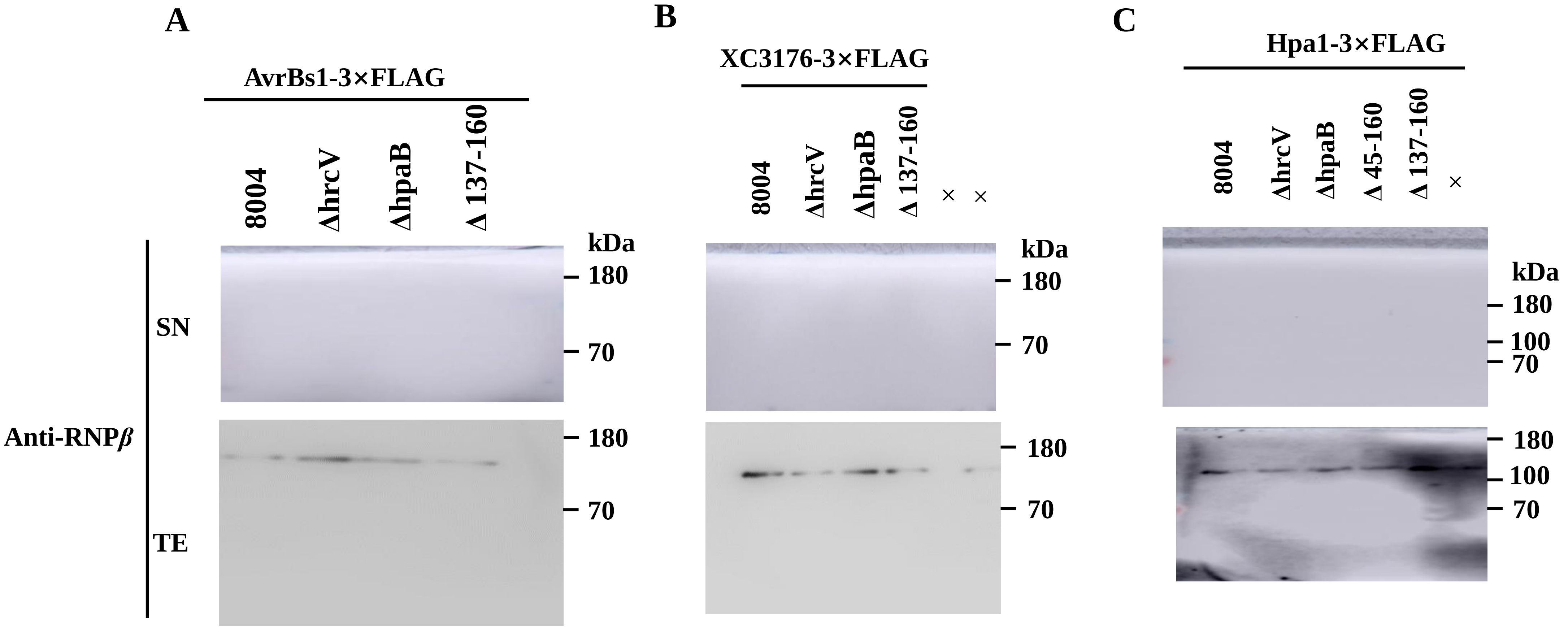

A
AvrBs1-3×FLAG
8004
ΔhrcV
ΔhpaB
Δ 137-160
kDa
180
70
SN
TE
Anti-RNPβ
B
XC3176-3×FLAG
8004
ΔhrcV
ΔhpaB
Δ 137-160
×
×
kDa
180
70
C
Hpa1-3×FLAG
8004
ΔhrcV
ΔhpaB
Δ 45-160
Δ 137-160
×
kDa
180
100
70

Figure 4A

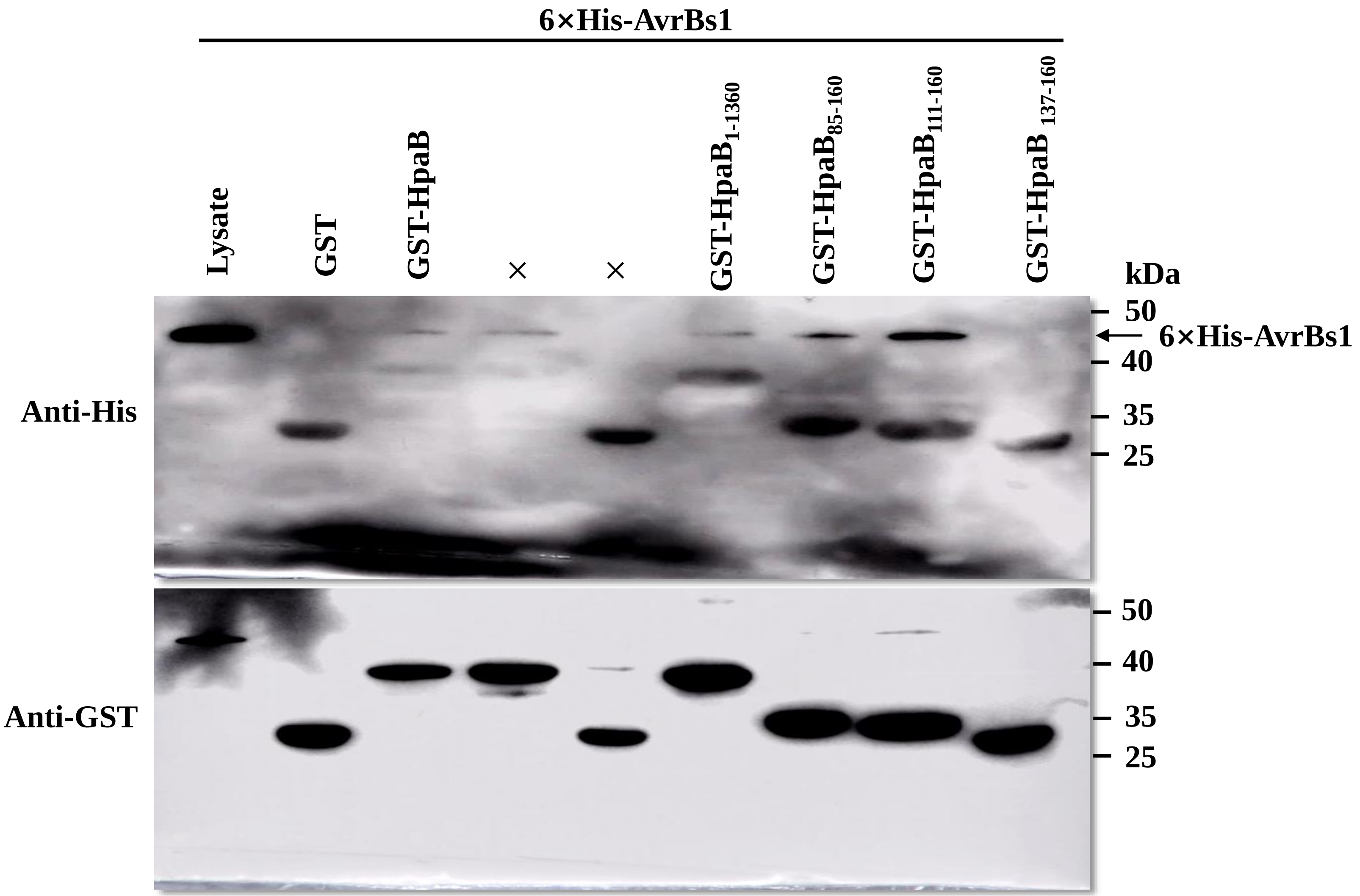
6×His-AvrBs1
Lysate
GST
GST-HpaB
×
×
GST-HpaB1-1360
GST-HpaB85-160
GST-HpaB111-160
GST-HpaB 137-160
kDa
50
40
35
25
6×His-AvrBs1
Anti-His
Anti-GST
50
40
35
25

Figure 4B

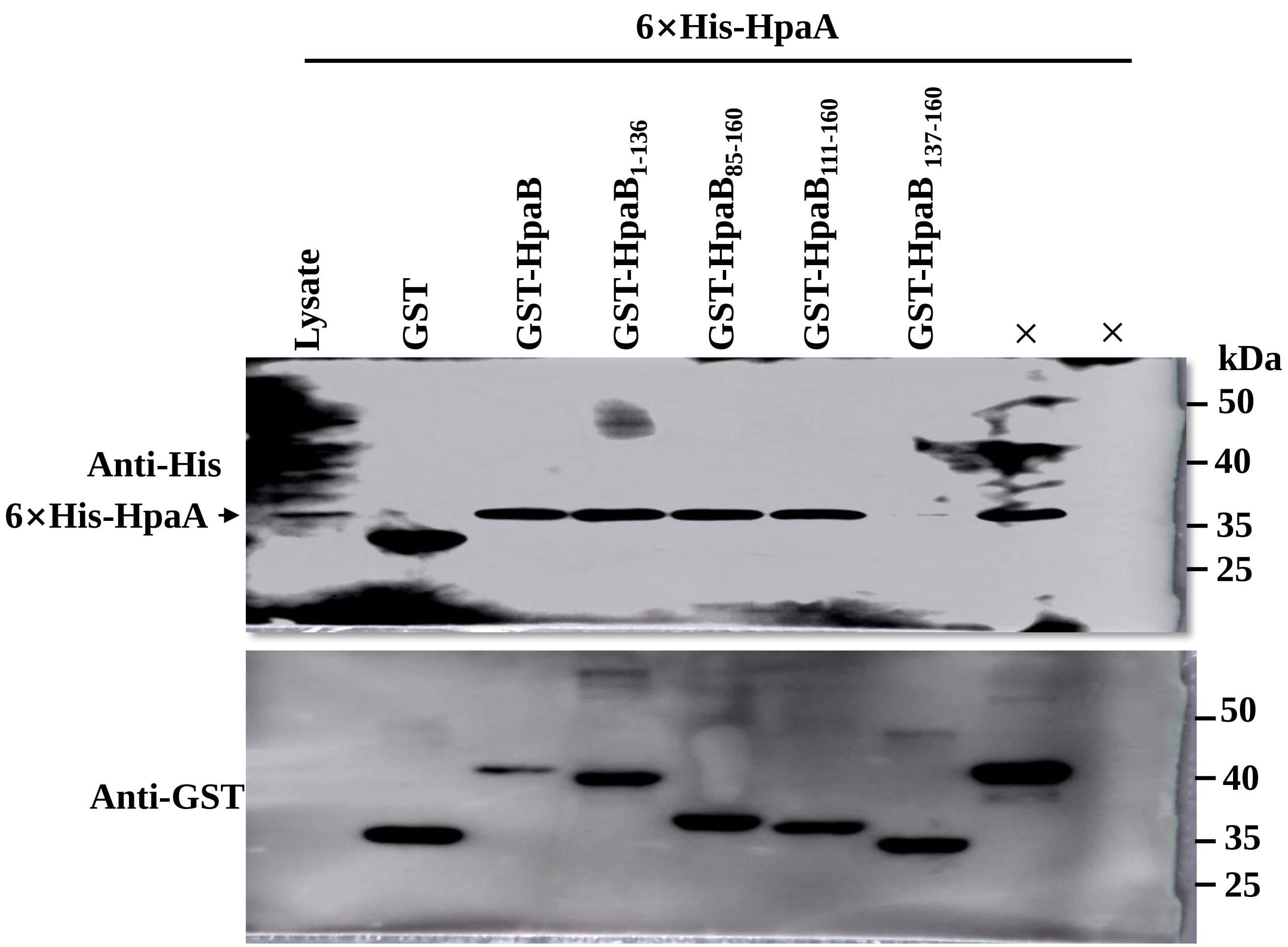

6×His-HpaA
Lysate
GST
GST-HpaB
GST-HpaB1-136
GST-HpaB85-160
GST-HpaB111-160
GST-HpaB 137-160
×
×
kDa
50
40
35
25
Anti-His
6×His-HpaA
Anti-GST
50
40
35
25

Figure 5A

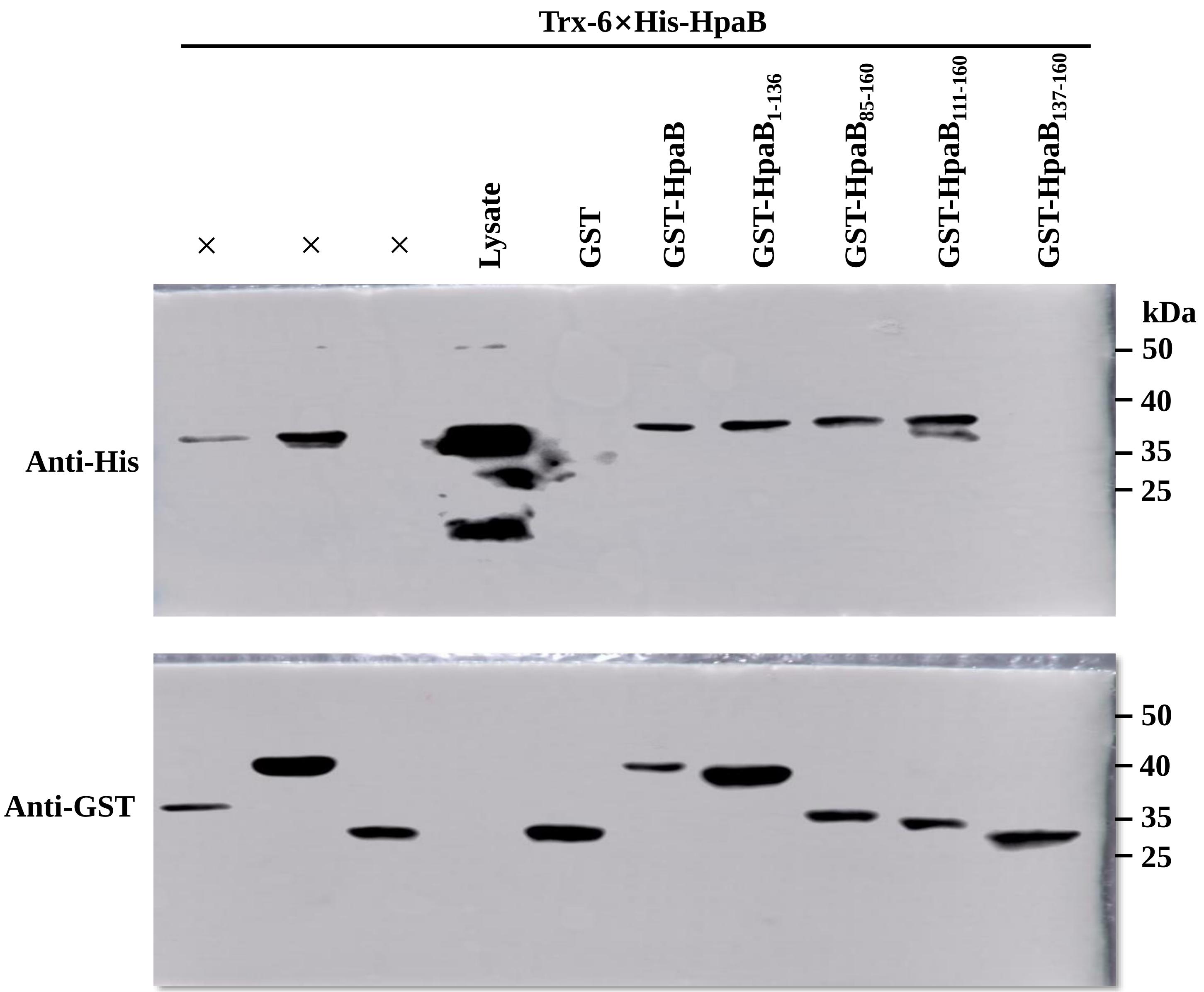
Trx-6×His-HpaB
×
×
×
Lysate
GST
GST-HpaB
GST-HpaB$_{1-136}$
GST-HpaB$_{85-160}$
GST-HpaB$_{111-160}$
GST-HpaB$_{137-160}$
kDa
50
40
35
25
Anti-His
50
40
35
25
Anti-GST

Figure 5B

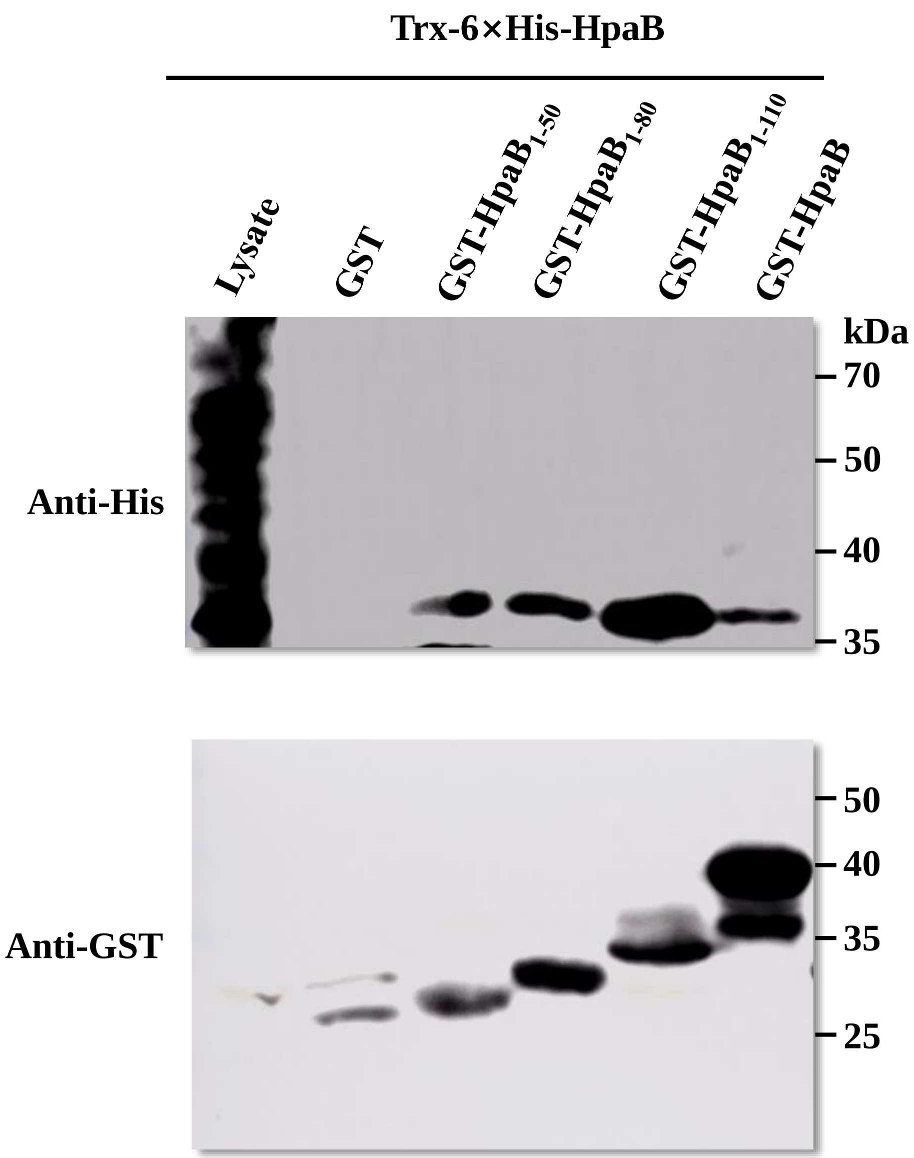

Figure 5C

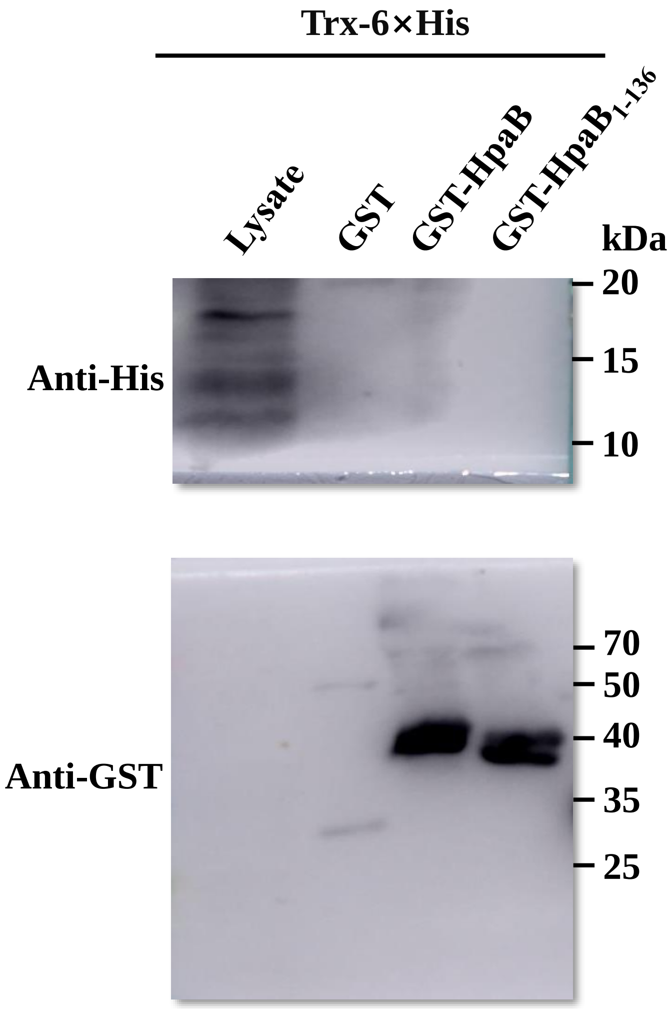

Figure 5D

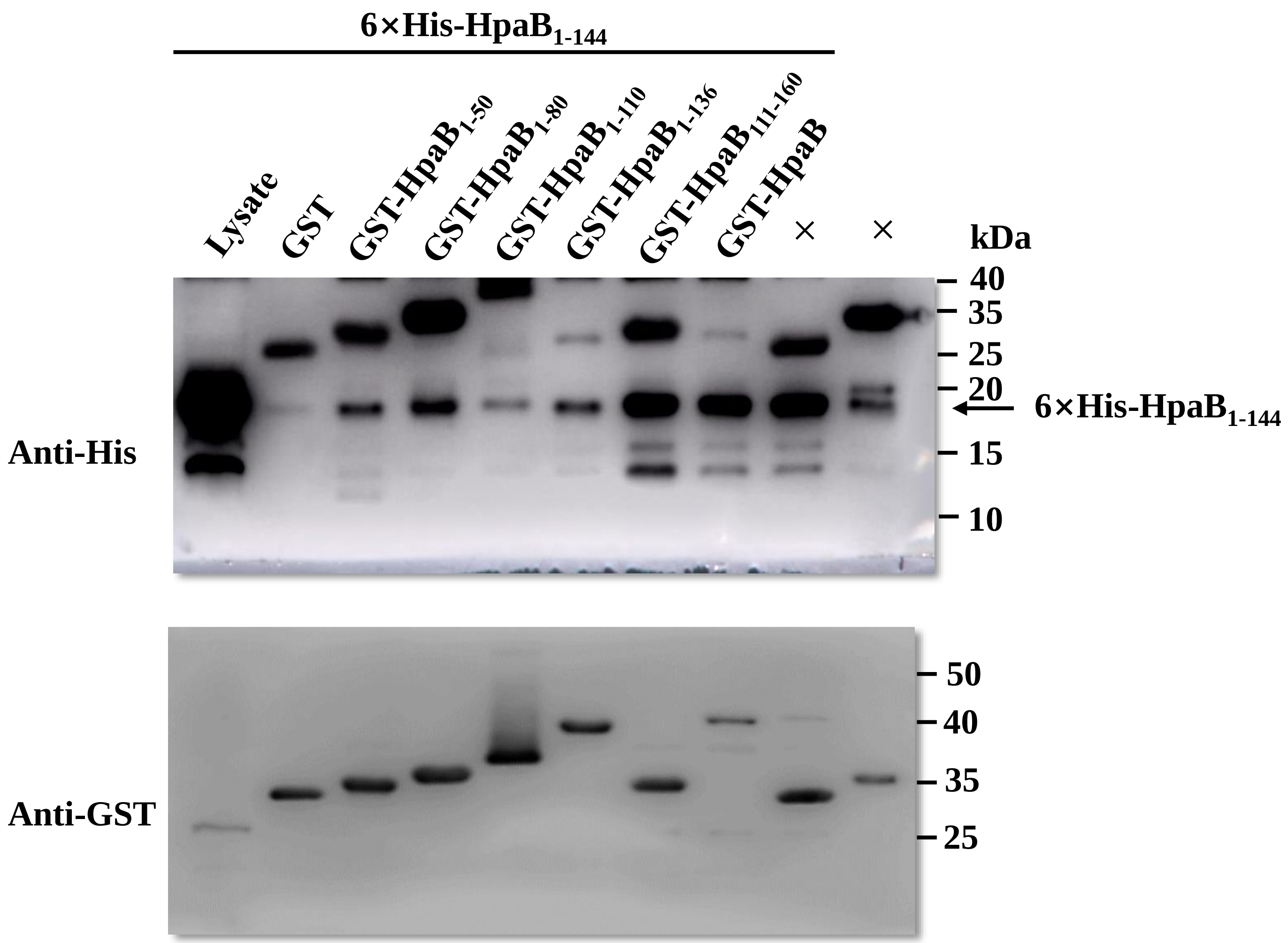

6×His-HpaB1-144
Lysate
GST
GST-HpaB1-50
GST-HpaB1-80
GST-HpaB1-110
GST-HpaB1-136
GST-HpaB111-160
GST-HpaB
×
×
kDa
40
35
25
20
15
10
6×His-HpaB1-144
Anti-His
Anti-GST
50
40
35
25

Figure 6

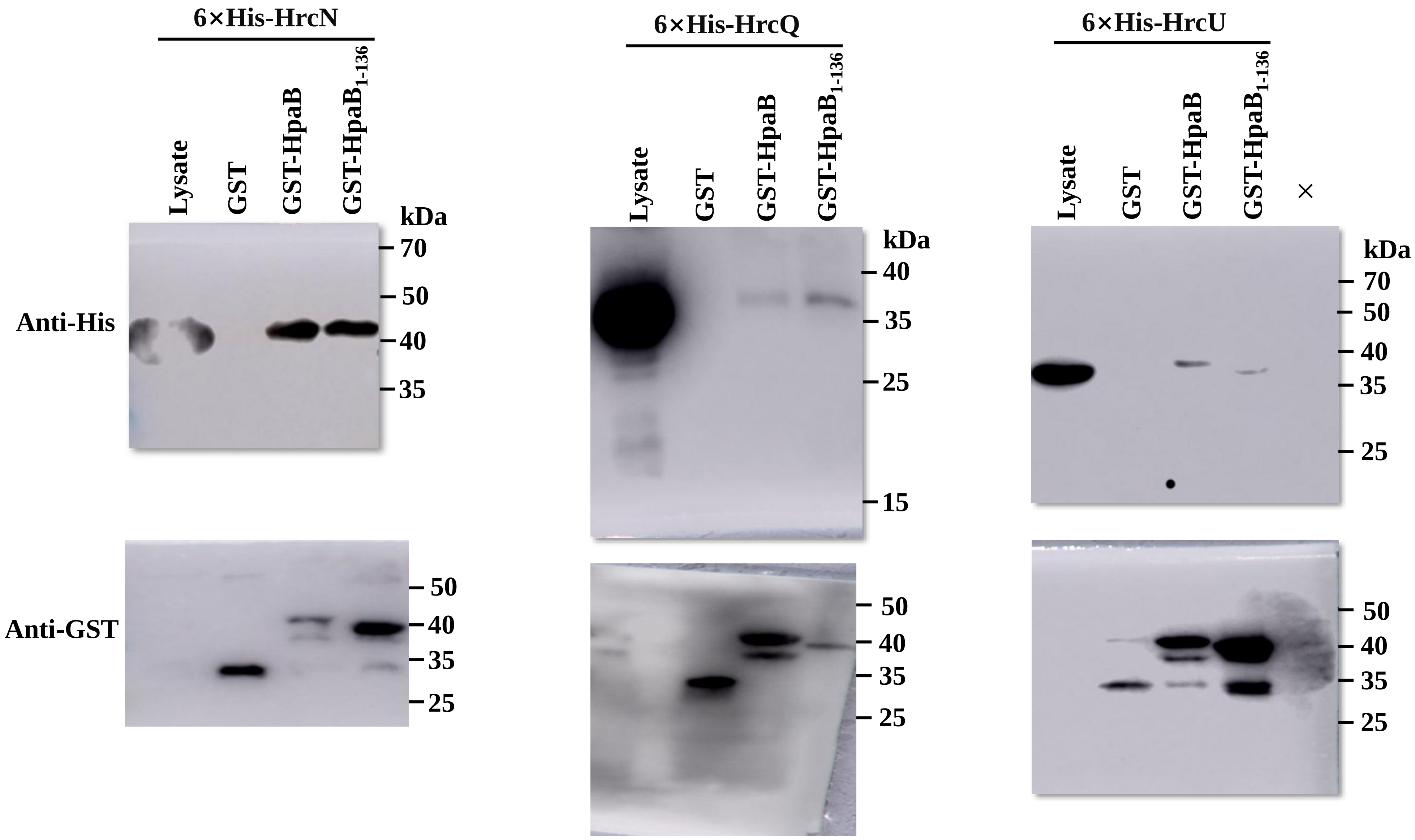

6×His-HrcN
6×His-HrcQ
6×His-HrcU
Lysate
GST
GST-HpaB
GST-HpaB1-136
×
kDa
Anti-His
Anti-GST
70
50
40
35
25
15

Figure 8A

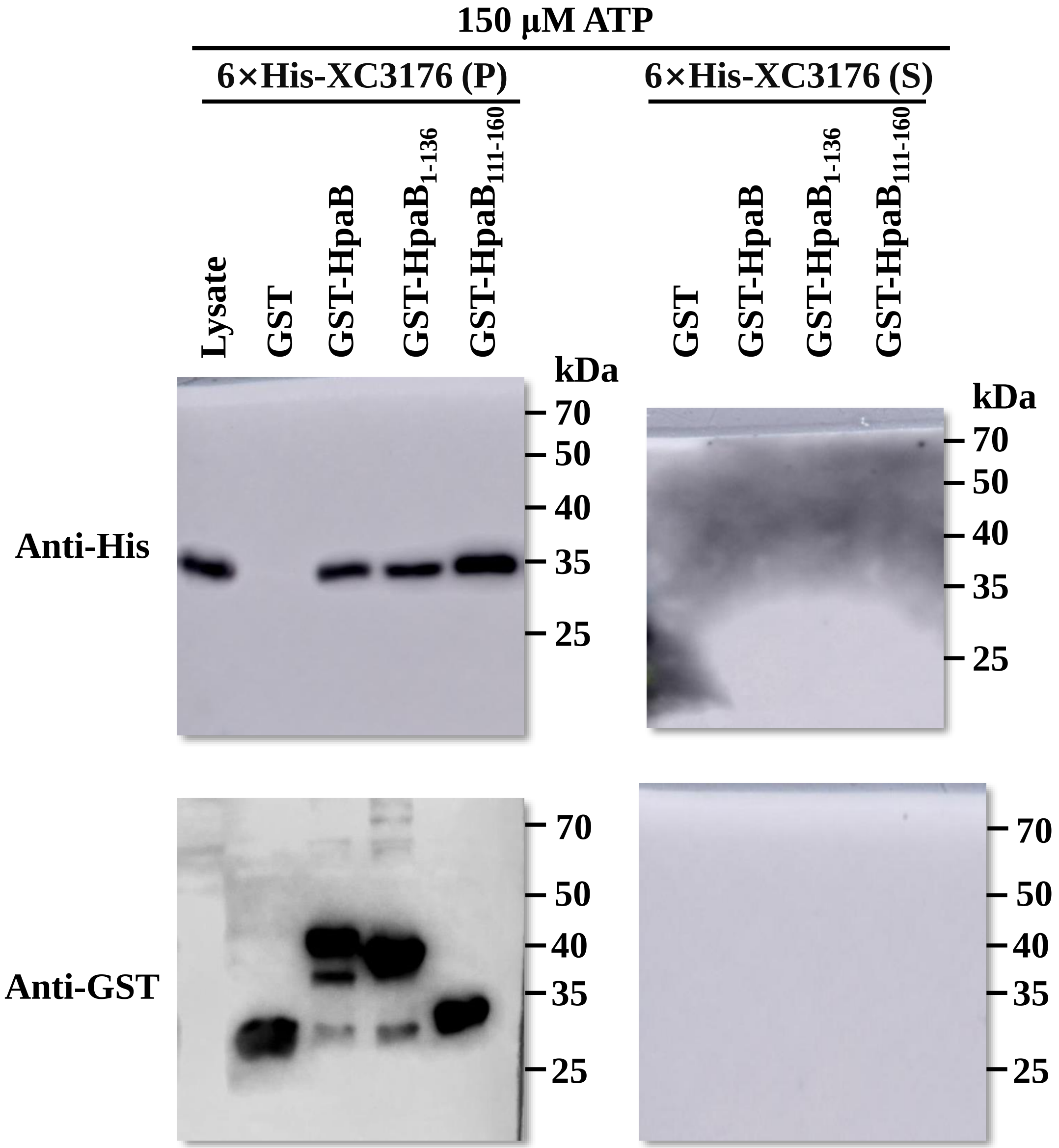

Figure 8B

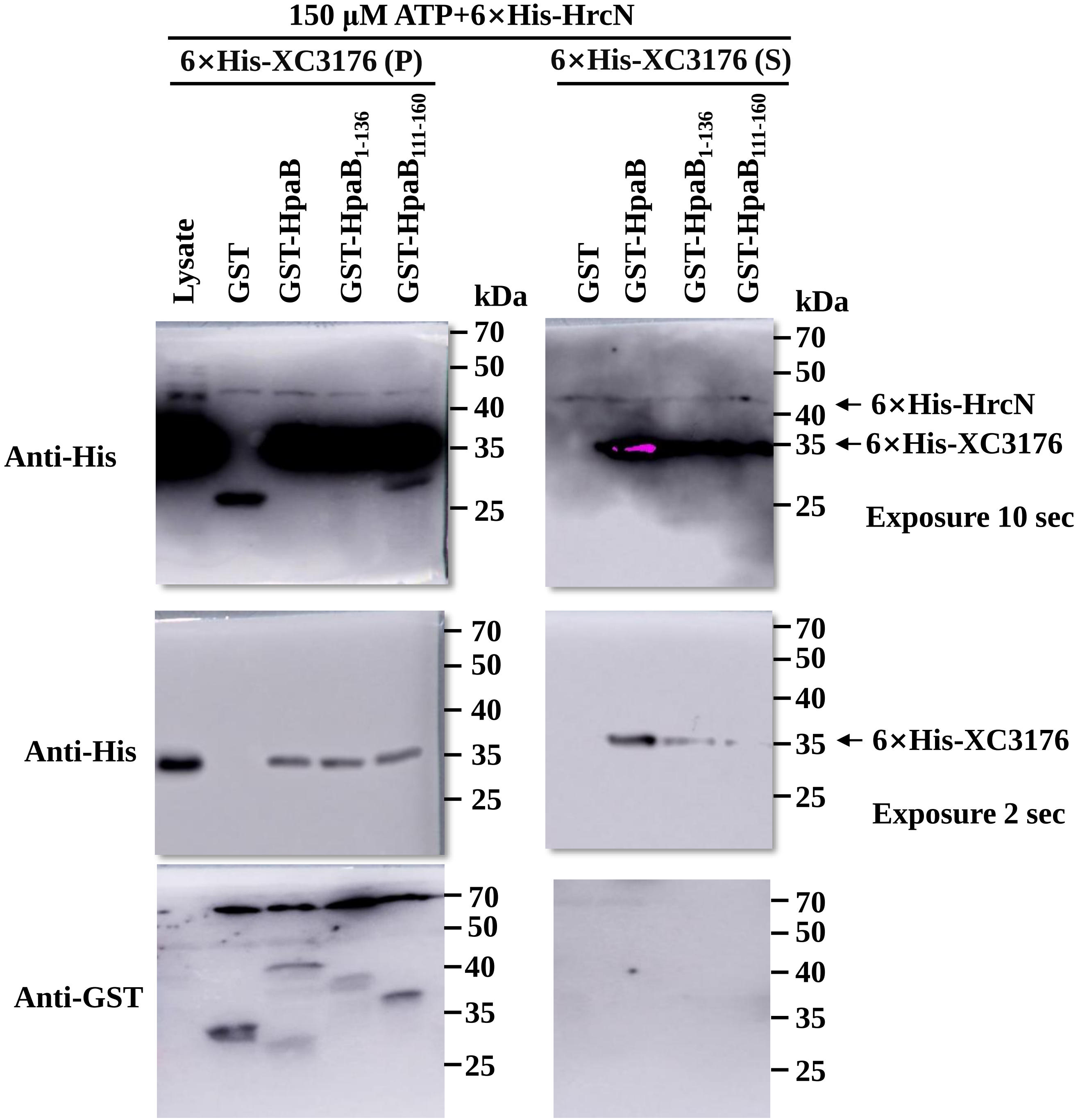

Figure 8C

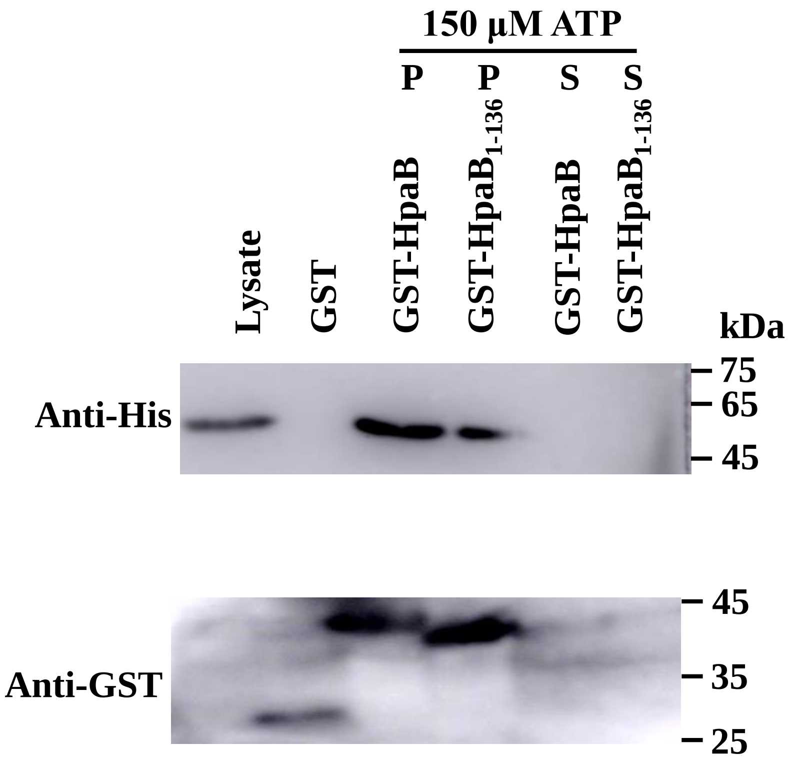

Figure 8D

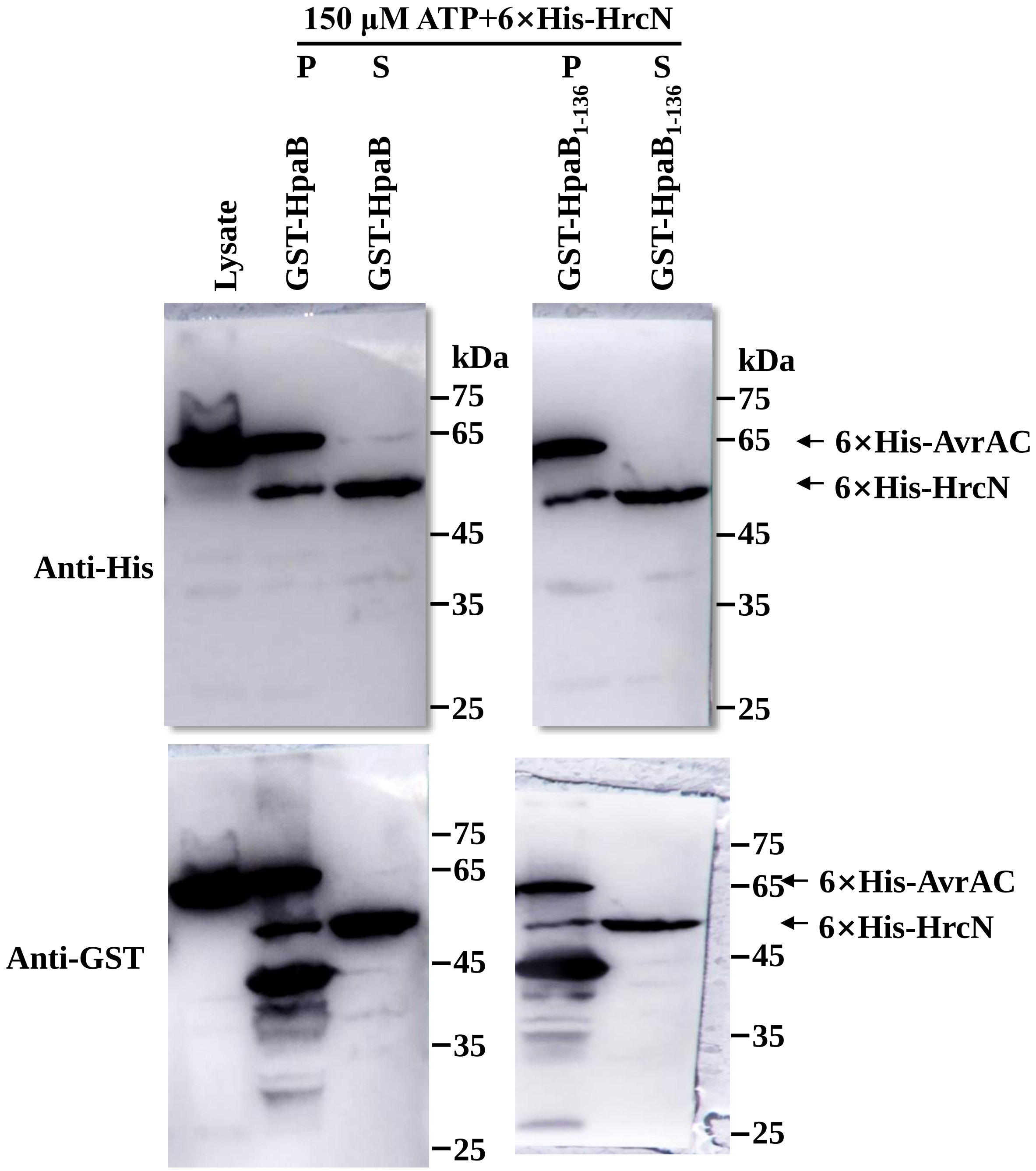

Figure S2A

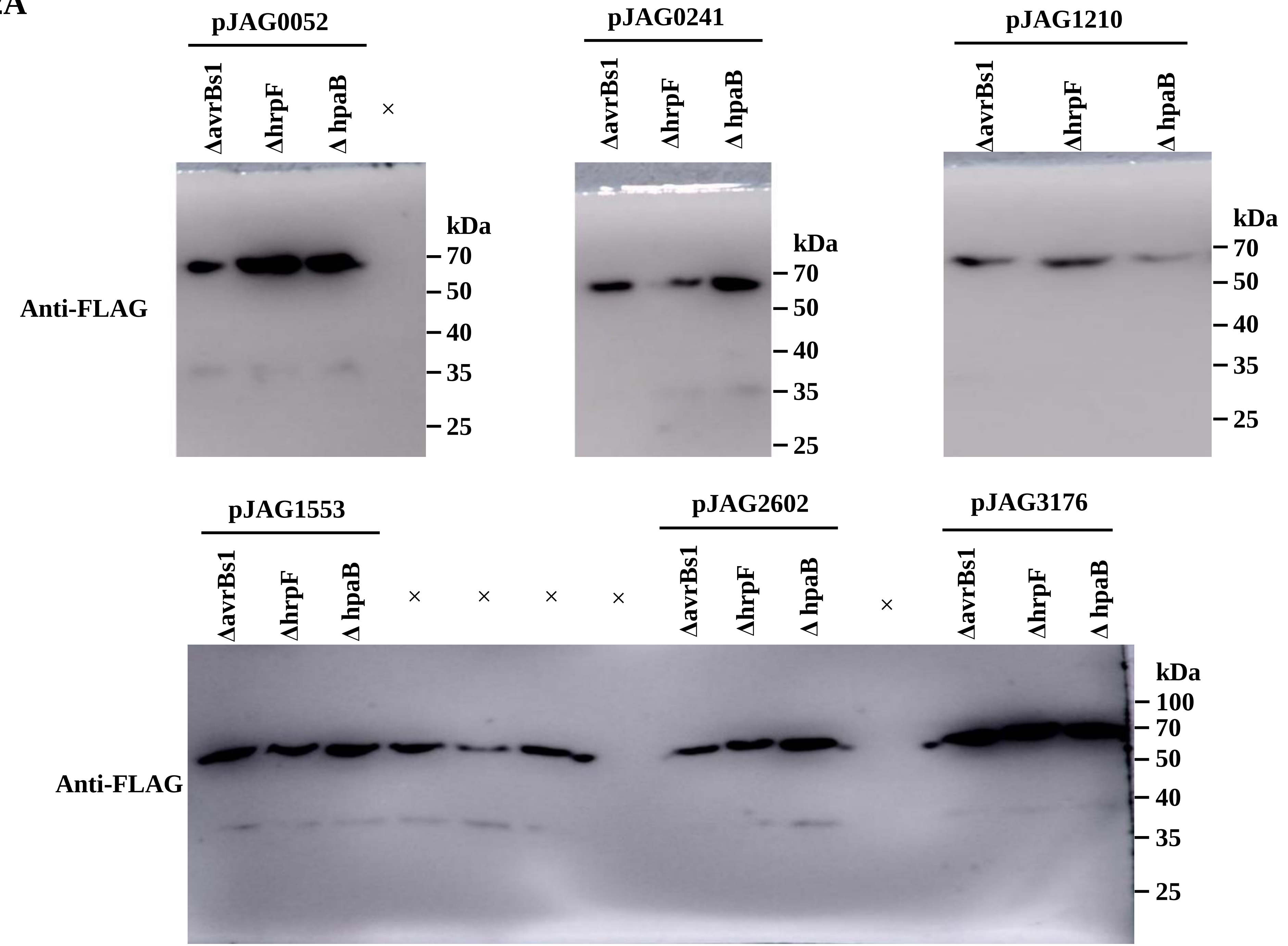
pJAG0052
ΔavrBs1
ΔhrpF
Δ hpaB
×
kDa
70
50
40
35
25
Anti-FLAG
pJAG0241
ΔavrBs1
ΔhrpF
Δ hpaB
kDa
70
50
40
35
25
pJAG1210
ΔavrBs1
ΔhrpF
Δ hpaB
kDa
70
50
40
35
25
pJAG1553
ΔavrBs1
ΔhrpF
Δ hpaB
× × × ×
pJAG2602
ΔavrBs1
ΔhrpF
Δ hpaB
×
pJAG3176
ΔavrBs1
ΔhrpF
Δ hpaB
kDa
100
70
50
40
35
25
Anti-FLAG

Figure S2B

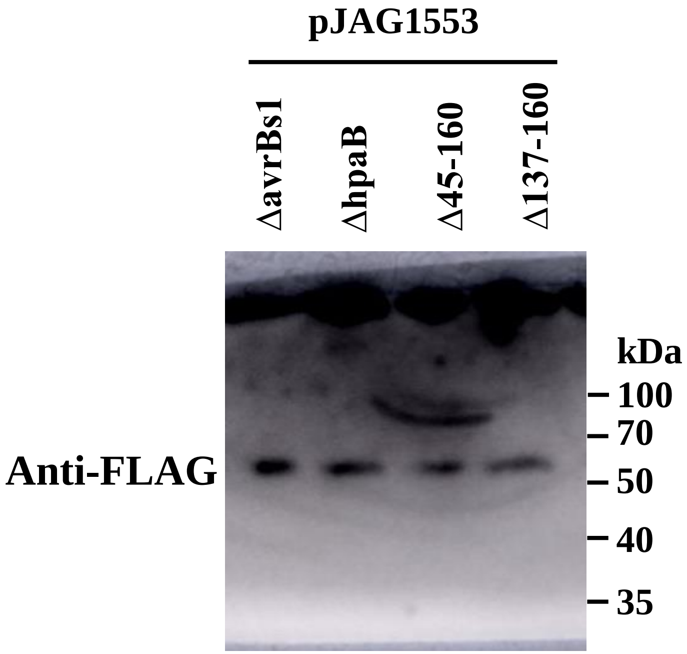

Figure S2C

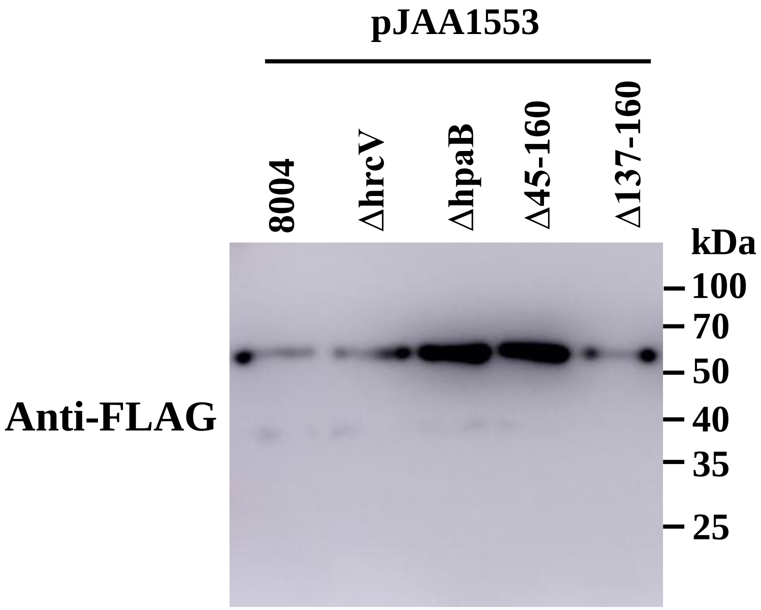

Figure S3

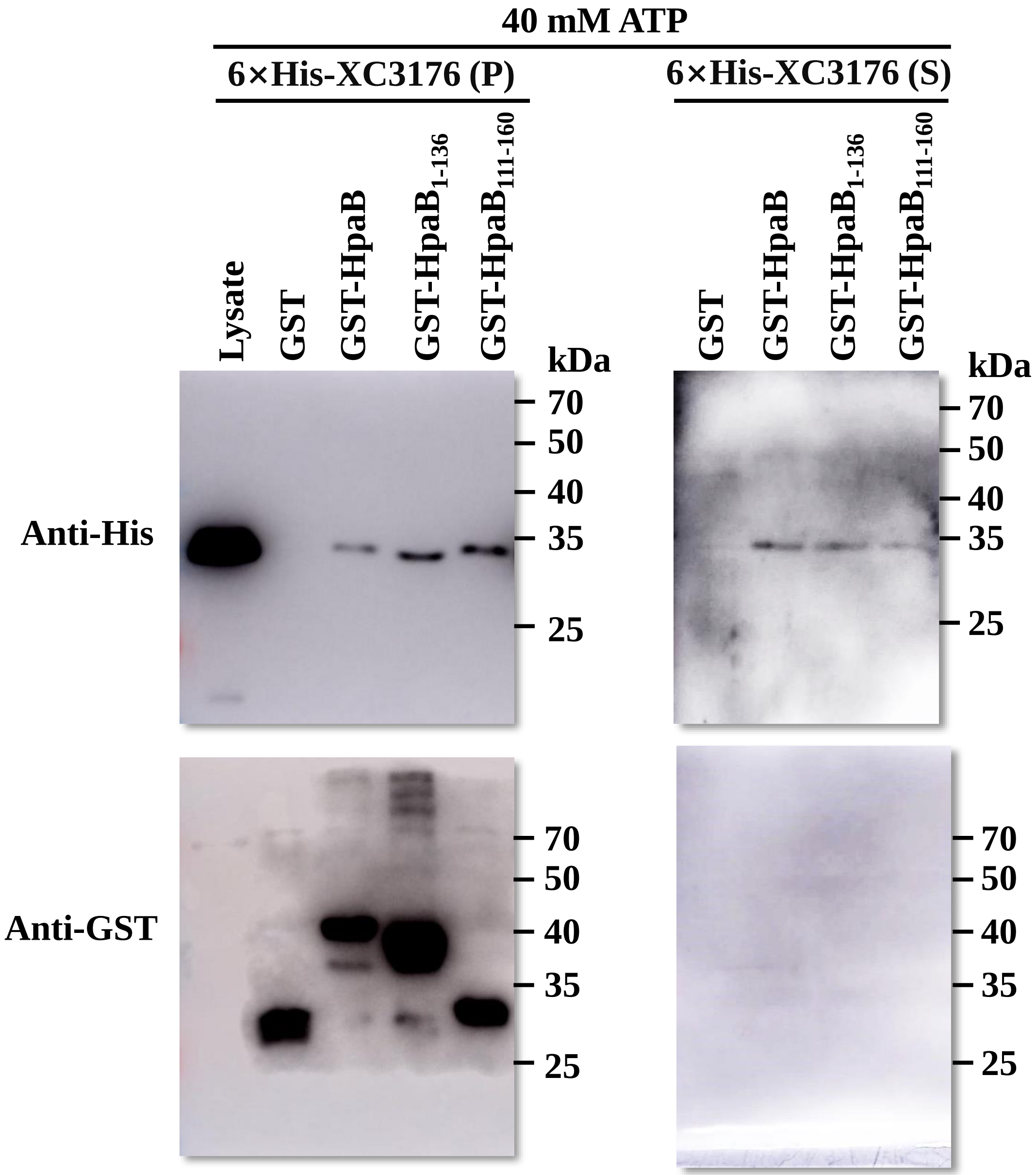

40 mM ATP
6×His-XC3176 (P)
6×His-XC3176 (S)
Lysate
GST
GST-HpaB
GST-HpaB1-136
GST-HpaB111-160
GST
GST-HpaB
GST-HpaB1-136
GST-HpaB111-160
kDa
70
50
40
35
25
kDa
70
50
40
35
25
Anti-His
Anti-GST
70
50
40
35
25
70
50
40
35
25
